# The Cancer Cell Metabolic Reprogramming Remodels the Tumor Microenvironment: Molecular Mechanisms and Therapeutic Strategies

**DOI:** 10.1002/advs.77424

**Published:** 2026-08-31

**Authors:** Guoqing Xiang, Zhicheng Zhou, Xue Jiang, Zhenguang Du, Yangyang Zhan, Xinli Liu

**Affiliations:** ^1^ Department of Endoscopy Cancer Hospital of Dalian University of Technology Liaoning Cancer Hospital & Institute Shenyang Liaoning Province China; ^2^ Department of Interventional Medicine Liaoning Provincial People's Hospital Shenyang Liaoning Province China; ^3^ Department of Pharmacy Shanghai Eastern Hepatobiliary Surgery Hospital Navy Military Medical University Shanghai China; ^4^ Department of Oncology Shanghai East Hospital Tongji University School of Medicine Shanghai China; ^5^ Department of Digestive Oncology Cancer Hospital of Dalian University of Technology Liaoning Cancer Hospital & Institute Shenyang Liaoning Province China

**Keywords:** cancer immune escape, cancer metabolic reprogramming, targeted therapeutic strategy, tumor microenvironment

## Abstract

Metabolic reprogramming and immune evasion are two key mechanisms that facilitate tumor progression. Tumor cells achieve their energy requirements for rapid proliferation through metabolic reprogramming, such as glucose metabolism, lipid metabolism, and amino acid metabolism. Tumor cell metabolic reprogramming can influence immune cell function in the tumor microenvironment through various mechanisms, ultimately facilitating tumor immune escape. Targeting tumor cell metabolism and combining metabolic regulation with immunotherapy can enhance anti‐tumor immune cells, inhibit the function of immunosuppressive cells, improve anti‐tumor therapy, and overcome immune escape. This review elucidates the regulatory mechanisms by which glucose, lipids, amino acid metabolism, nucleotide metabolism, oxidative phosphorylation, lactate, and hypoxia modulate the proliferation, differentiation, and function of anti‐tumor immune cells and immunosuppressive cells. Regulatory strategies to restore the anti‐tumor function of immune cells in response to metabolic changes were discussed. These strategies are expected to improve the effect of immunotherapy and are used in combination with other treatments to provide new ideas for cancer management.

## Graphical abstract

This review elucidates how cancer cell metabolic reprogramming—across glucose, lipid, amino acid, and nucleotide pathways—remodels the tumor microenvironment to suppress anti‐tumor immunity and promote immune escape. Targeting these metabolic axes offers promising strategies to overcome immunotherapy resistance and enhance cancer treatment. Figure created in https://BioRender.com.

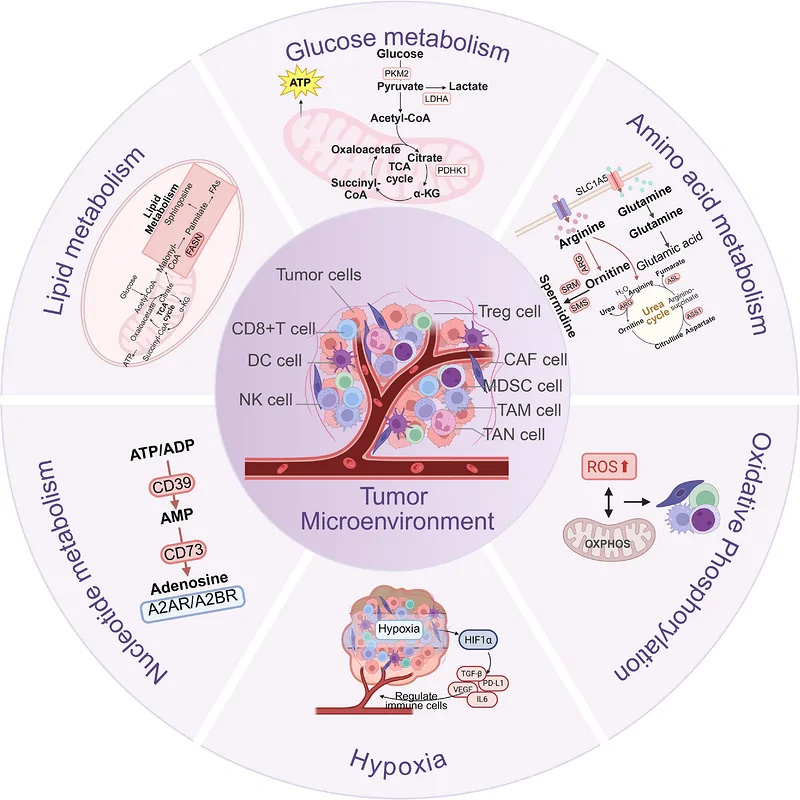

## 1 Introduction

Immune escape is one of the top ten features of malignant tumors. Although tumor cells can initially be identified and attacked by the immune system, they eventually escape immune surveillance through a process known as immune editing, allowing them to survive, proliferate, and metastasize. The mechanisms underlying tumor immune escape are highly complex. In addition to the intrinsic properties of tumor cells, immune‐suppressive components within the tumor microenvironment (TME) play indispensable roles in facilitating immune escape. There are various kinds of immune cells in TME, including anti‐tumor immune cells and immunosuppressive cells [1]. Anti‐tumor immune cells are a class of cells that can recognize foreign antigens, pathogens, or abnormal cells (such as tumor cells) and stimulate or enhance the immune response by directly killing, secreting cytokines, or activating other immune cells, including CD8^+^ T cells, natural killer (NK) cells, and dendritic (DC) cells. However, they may indirectly promote immune escape due to exhaustion, phenotypic switching, differentiation, or regulation in chronic stimulation or specific microenvironments [2]. For example, metabolic reprogramming in tumor cells suppresses CD8^+^T cell activity, causing them to transition to a functionally “exhausted” phenotype‐exhausted CD8^+^T cells (Tex). Tex cells exhibit impaired self‐renewal capacity and progressive loss of effector function, accompanied by multiple metabolic defects: inhibited glycolysis, disrupted lipid metabolism, and accumulation of dysfunctional mitochondria [3, 4]. Immune‐suppressive cells are a category of cells that assist tumor cells in evading immune clearance by inhibiting effector immune cells, interfering with antigen presentation, and regulating metabolism. They include immune cells that directly exert suppressive functions, such as regulatory T cells (Tregs), tumor‐associated macrophages (TAMs), tumor‐associated neutrophils (TANs), myeloid‐derived suppressor cells (MDSCs), and stromal cells that indirectly suppress antitumor immune responses by remodeling the microenvironment, such as cancer‐associated fibroblasts (CAFs) [5]. As the most abundant stromal cells in the tumor microenvironment, CAFs are increasingly recognized as active modulators of immune activity through antigen presentation and cytokine secretion, ultimately promoting tumor progression [6]. In this review, we describe CAFs as part of the immunosuppressive cell population.

Tumor cells often adapt to the high nutritional demands of rapid proliferation and survival by reprogramming their metabolic pathways‐a process termed metabolic reprogramming. Unlike normal cells, which primarily rely on efficient aerobic oxidation for energy production, tumor cells prefer faster yet less efficient metabolic modes, such as aerobic glycolysis, abnormal lipid metabolism, enhanced glutamine utilization, and mitochondrial remodeling. Metabolic reprogramming enables tumor cells to meet biosynthetic demands, survive in hypoxic environments, and resist oxidative stress. More importantly, these metabolic alterations profoundly reshape the function of immune cells within the tumor microenvironment, directly or indirectly suppressing antitumor immunity and promoting immune escape [7, 8]. For example, in environments characterized by high glycolytic activity and lactic acid accumulation, the effector functions of anti‐tumor immune cells are impaired, enabling tumor cells to evade immune surveillance. Conversely, immune‐suppressive cells often maintain or enhance their functions under such metabolic reprogramming condition [9].

**FIGURE 1:**
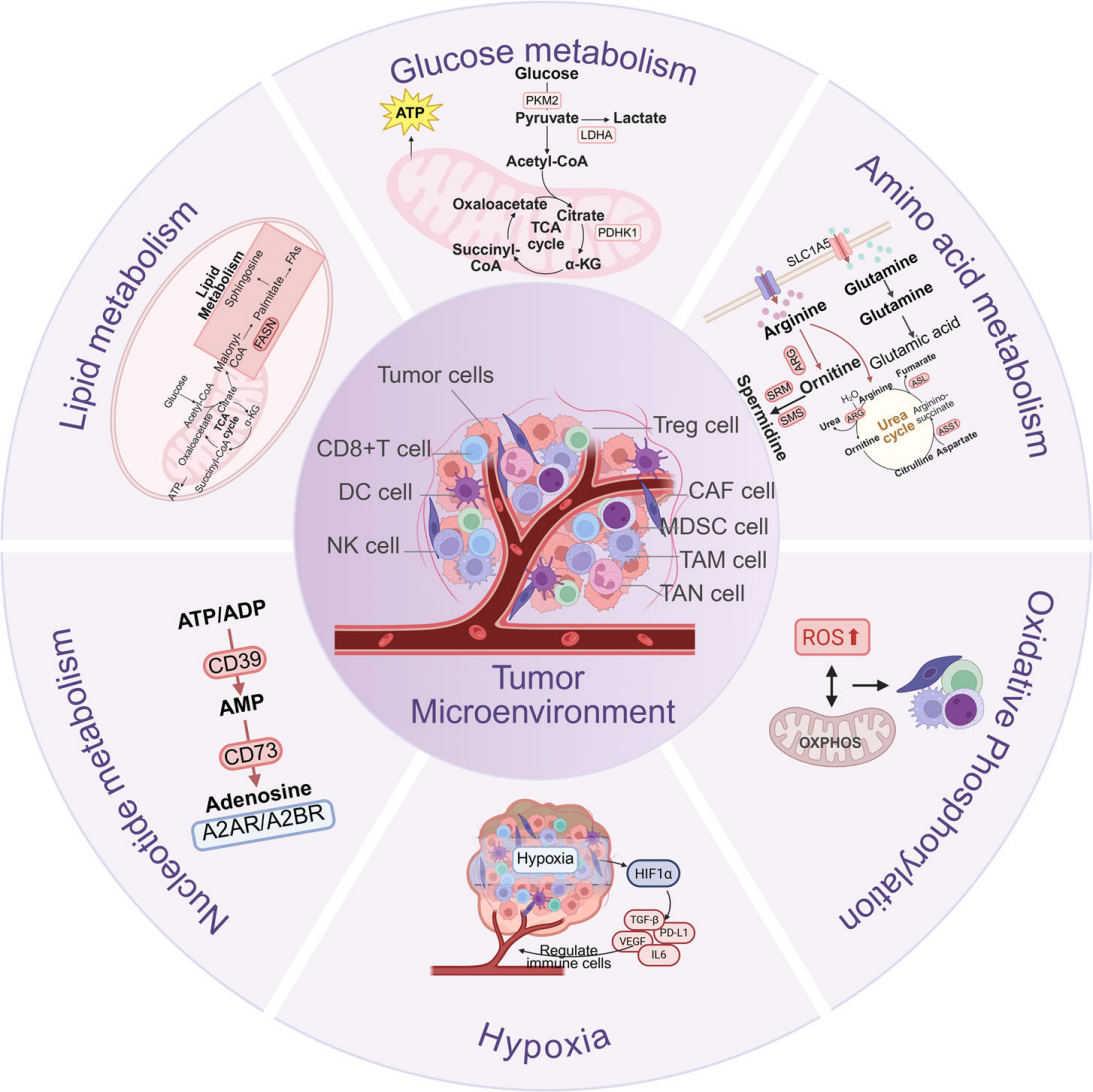
Regulatory Mechanisms of Tumor Metabolism on Immune Cells. Tumor cells influence immune cell function within the tumor microenvironment through metabolic reprogramming (such as glucose metabolism, lipid metabolism, amino acid metabolism, nucleotide metabolism, oxidative phosphorylation, and hypoxia), thereby promoting immune evasion and tumor progression. The key metabolic pathway in glucose metabolism is glycolysis. Competitive glucose uptake by tumor cells and immune cells leads to glucose deprivation in the TME, causing lactic acid accumulation and thereby promoting immune evasion. Lipid metabolism primarily induces immunosuppression through the accumulation of metabolites like lipids and the activation of metabolic enzymes. Amino acids such as glutamine, arginine, and tryptophan disrupt immune function by regulating signaling pathways and metabolic enzymes, inducing diverse immune cell responses. Nucleotide metabolism primarily promotes inflammation and anti‐tumor immunity through the purine‐based immunosuppressive pathway involving CD39‐CD73‐adenosine. Excessive mitochondrial oxidative phosphorylation and ROS production, along with hypoxia‐induced HIF1α upregulation, are all closely associated with TME immunosuppression. Created in https://BioRender.com.

This review aims to summarize recent advances in understanding how tumor‐intrinsic metabolic reprogramming remodels the TME to promote immune escape, and to discuss the current progress and potential of metabolism‐targeted therapeutic strategies in enhancing cancer immunotherapy (Figure 1).

## 2 Regulatory Mechanisms and Therapeutic Strategies of Glucose Metabolism on Immune Cells

**FIGURE 2:**
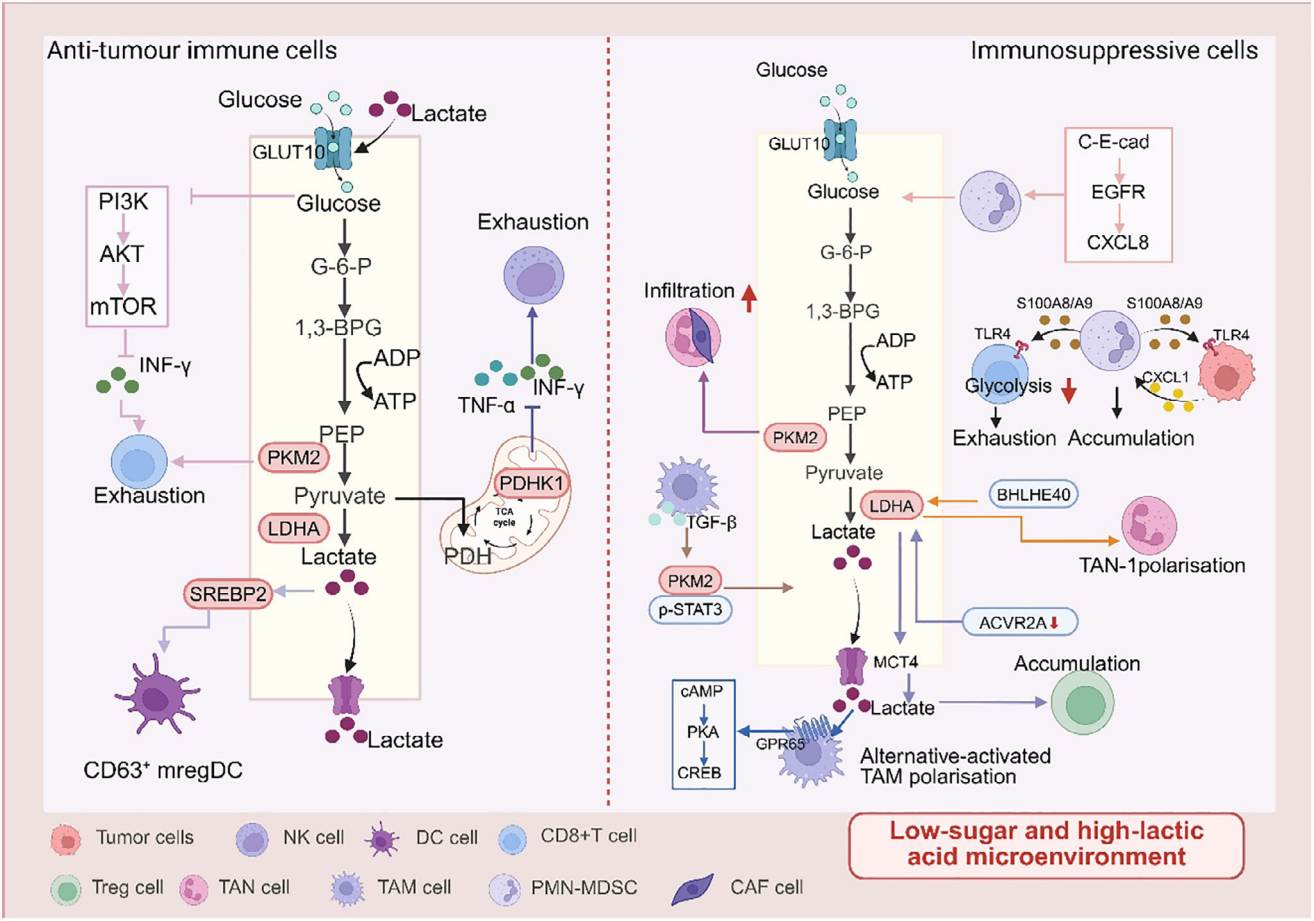
Mechanisms of glucose metabolism regulating immune cells. When immune cells are exposed to low glucose and high glycolysis environments, the functions of antitumor immune cells such as CD8^+^T cells, DCs, and NK cells are suppressed, leading to an exhausted state. Conversely, immune‐suppressive cells like TANs, TAMs, and MDSCs are recruited, enhancing immunosuppression. Abbreviations: PKM2: Pyruvate Kinase M2; LDHA: Lactate Dehydrogenase A; PDHK1: Pyruvate Dehydrogenase Kinase 1; G‐6‐P: Glucose‐6‐Phosphate; 1,3‐BPG: 1,3‐Bisphosphoglycerate; PEP: Phosphoenolpyruvate; PDH: Pyruvate Dehydrogenase complex; SREBP2: Sterol Regulatory Element‐Binding Protein 2; BHLHE40: Basic Helix‐Loop‐Helix Family Member E40; ACVR2A: Activin A Receptor, Type II A. Created in https://BioRender.com.

Glucose metabolism not only drives the malignant proliferation of cancer cells themselves but also profoundly shapes the fate of the immune microenvironment. Within TME, glucose metabolism serves as the primary metabolic fuel. Tumor cells efficiently consume glucose and produce excessive lactate by upregulating glucose transporters and aerobic glycolysis, thereby depleting glucose in the TME and creating a low‐glucose, high‐lactate environment. This environment induces distinct functional changes in immune cells: anti‐tumor immune cells become functionally exhausted, while immune‐suppressive cells adapt to thrive in this environment, gaining a survival advantage (Figure 2).

### 2.1 Regulatory Mechanisms and Therapeutic Strategies of Glucose Metabolism in Counteracting Anti‐tumor Immune Cells

One of the most central pathways in glucose metabolism is glycolysis, which plays a crucial role in regulating antitumor immune cells. When CD8^+^T cells face glucose deprivation, it inhibits the mTOR signaling pathway and reduces IFN‐γ production. Together, these two effects suppress glycolysis and lead to partial T cell exhaustion [10, 11]. Pyruvate kinase M2 (PKM2), as the rate‐limiting enzyme catalyzing the final step of glycolysis, has been identified as a critical metabolic node. High PKM2 expression correlates with CD8^+^T cell fate. PKM2 deficiency redirects metabolism toward the pentose phosphate pathway (PPP), altering CD8^+^T cell state and preventing terminal exhaustion [12]. Another key enzyme, pyruvate dehydrogenase kinase 1 (PDHK1), also functions as a factor limiting the activity of anti‐tumor immune cells. Highly expressed in the tumor microenvironment (TME), PDHK1 exerts its effects by suppressing cytokine secretion and inducing NK cell exhaustion. Inhibiting PDHK1 can reactivate exhausted NK cells within the TME, thereby restoring their function and enhancing anti‐tumor immune responses [13]. The highly glycolytic phenotype of tumor cells competes with dendritic cells (DCs) for glucose, placing DCs in a metabolic crisis. This impairs DC maturation, reduces their migratory capacity, and diminishes their ability to activate T cells. Research reports indicate that fatty acid‐conjugated alpha‐fetoprotein (AFP) redirects human dendritic cell metabolism toward glycolysis, thereby suppressing immune stimulation and promoting immunosuppression [14]. Therefore, targeting glycolytic metabolism within the tumor microenvironment represents an effective strategy for restoring dendritic cell function. However, studies have revealed that synergistically enhancing dendritic cell glycolysis or concurrently activating STING can amplify antitumor immune responses, offering novel strategies to improve cancer immunotherapy. This occurs because enhanced glycolysis within dendritic cells generates substantial ATP, which directly activates the STING pathway. STING activation subsequently upregulates hypoxia‐inducible factor (HIF1α), further accelerating glycolysis and forming a positive feedback loop that enhances the antitumor activity of dendritic cells [15, 16, 17].

To enhance the function of anti‐tumor immune cells, targeting glycolysis represents a highly significant therapeutic strategy, primarily achievable through inhibiting glucose transporters, key enzymes in the glycolytic pathway, and glycolysis inhibitors. Research indicates that lactate binding to glucose transporter 10 (GLUT10) suppresses glucose uptake and weakens CD8^+^T cell activation. Based on this, Liu et al. [18] developed the mimetic peptide PG10.3, which enhances antitumor efficacy by disrupting the binding between lactate and GLUT10, thereby promoting glucose utilization by CD8^+^T cells. Furthermore, combination therapy with PG10.3 and GLUT10 inhibitors or anti‐programmed death‐1 (anti‐PD‐1) antibodies demonstrated synergistic antitumor effects. Modulating the activity of key glycolytic enzymes can also reshape the metabolic profiles of antitumor immune cells, enhancing their immune activation capacity. For example, overexpression of fructose‐1,6‐bisphosphatase 1 (FBP1) inhibits glycolysis in tumor cells, activates the cGAS/STING/NF‐κB pathway, promotes IL‐33 secretion, and consequently enhances CD8^+^T cell function and dendritic cell activation [19]. Similarly, inhibiting tumor glycolysis with the PFKFB3 inhibitor PFK15 while supplementing dendritic cells (DCs) with fructose‐1,6‐bisphosphate (F16BP) restores DC metabolic capacity and antigen presentation function. Inamdar et al. [20] developed a metabolically compensatory vaccine containing F16BP for DC adoptive therapy. This vaccine selectively enhances DC activity under glycolysis‐inhibited conditions and induces robust Tc1/Tc17 responses, achieving synergistic suppression of tumor metabolism and enhanced antitumor immunity. Furthermore, 11β‐hydroxysteroid dehydrogenase type 1 (11β ‐HSD1) is associated with NK cell immune evasion and regulates hepatic glucose output. Studies indicate that 11β‐HSD1 inhibitors reduce fibrotic area, alanine aminotransferase (ALT), and aspartate aminotransferase (AST) levels in a mouse liver fibrosis model. They alleviate liver fibrosis by inhibiting the Notch pathway and enhancing NK cell‐mediated immune responses [21]. Low‐dose glycolysis inhibitor 2‐deoxy‐D‐glucose (2DG) significantly enhances antitumor immune cell function and in vivo antitumor efficacy by inducing metabolic stress and epigenetic reprogramming [22]. Sasaki et al. [23] designed a nanoparticle‐mediated glucose analog‐2‐deoxyglucose (2DG)‐encapsulated within poly (lactic‐co‐glycolic acid) nanoparticles (2DG‐PLGA‐NPs). This nanoparticle therapy reduced lactate production in hepatocellular tumors, increased interferon‐γ‐positive T cell numbers, and enhanced glucose uptake and CD8^+^T cell chemotaxis. Notably, 2DG‐PLGA‐NPs not only potentiates the antitumor effects of sorafenib or anti‐PD‐1 therapy but also overcomes PD‐1 resistance.

Additionally, the accumulation of lactate‐a glycolytic product‐inhibits the cytotoxicity of antitumor immune cells, thereby promoting immune escape. Research reveals that N‐Myc Downregulated Gene 1 (NDRG1) stabilizes lactate dehydrogenase A (LDHA) by inhibiting its ubiquitination, thereby enhancing glycolysis and increasing lactate accumulation. Excess lactate further impairs CD8^+^T cell function, upregulates immunosuppressive gene expression, and thus amplifies tumor‐induced immunosuppression [24]. Additionally, lactate activates the transcription factor SREBP2, driving the mevalonate biosynthesis pathway to induce conventional dendritic cells to differentiate into CD63+ mature regulatory dendritic cells (mregDCs) with immunosuppressive functions. This process subsequently inhibits antigen presentation and promotes Treg/Th2 differentiation, leading to immune tolerance [25]. However, studies have found that targeting the mTOR/AMPK metabolic pathway or blocking the monocarboxylate transporter 1 (MCT1) can reverse metabolic reprogramming in dendritic cells (DCs), restoring their antigen‐presenting function and T‐cell activation capacity. This offers a novel strategy for overcoming tumor immune escape through metabolic intervention [26]. To counteract the effects of lactate metabolic reprogramming on tumor cells, modulating lactate metabolism to reshape the immunosuppressive microenvironment has emerged as a potential strategy for cancer treatment. Liu et al. [27] developed a tumor‐selective nanodelivery system, PIMDQ/Syro‐RNP. This system simultaneously blocks lactate efflux by inhibiting Monocarboxylate Transporter 4 (MCT4), thereby reducing lactate levels in the tumor microenvironment (TME). This action relieves T‐cell suppression and induces tumor cell acidosis. Concurrently, it releases TLR7/8 agonist IMDQ to activate dendritic cells, synergistically promoting anti‐tumor T‐cell immune responses. This system offers an efficient metabolic‐immune synergistic intervention strategy to enhance the efficacy of immunotherapy.

### 2.2 Regulatory Mechanisms and Therapeutic Strategies of Glucose Metabolism in Immunosuppressive Cells

Unlike the dilemma faced by anti‐tumor immune cells in competing for glucose, immune‐suppressive cells rely on glycolysis for energy. They utilize the glycolytic pathway to sustain proliferation and obtain the fuel and mediators necessary for survival, while also indirectly exerting immunosuppressive functions by supplying energy to tumor cells.

Enhanced glycolysis in TME stabilizes the alternatively activated state of TAMs, suppressing immune surveillance and promoting tumorigenesis, while inhibiting glycolysis suppresses TAM function and restores antitumor immunity. For example, alternatively activated TAMs secrete TGF‐β, which promotes the formation of PKM2/p‐STAT3 nuclear complexes within bladder cancer cells, thereby enhancing glycolysis and PD‐L1 expression to drive immune evasion [28]. MDSCs are one of the primary immunosuppressive cell populations in the tumor microenvironment, with two major subpopulations currently identified: Polymorphonuclear myeloid‐derived suppressor cells (PMN‐MDSCs) and monocyte‐derived myeloid‐derived suppressor cells (M‐MDSCs) [29]. Their core characteristic lies in potent immune‐suppressive capabilities, particularly their ability to inhibit T cell proliferation and activity, thereby promoting tumor immune escape [30]. In gastric cancer, tumor cells recruit PMN‐MDSCs via the S100A8/A9‐TLR4‐CXCL1 axis. Activated PMN‐MDSCs suppress glycolysis and cytokine secretion in CD8^+^T cells through the S100A8/A9‐TLR4‐AKT‐mTOR pathway, inducing T cell exhaustion [31]. In breast cancer, the protein C‐E‐cad encoded by circular RNA recruits PMN‐MDSCs by activating the EGFR‐CXCL8 axis and maintains their glycolytic activity, thereby enhancing immunosuppression [32]. PKM2 is also closely associated with the activity of TAN and CAF within the tumor microenvironment (TME). PKM2 expression is upregulated by hypomethylation and m6A modification, enhancing glycolytic flux, angiogenesis, and epithelial‐mesenchymal transition while promoting CAF/TAN infiltration and PD‐L1 expression, thereby reshaping the immune landscape [33]. Research has revealed that transcription factor BHLHE40, acting as a downstream effector of hypoxia and endoplasmic reticulum stress, can directly activate the transcription of glycolytic genes such as LDHA. This promotes the polarization of tumor‐associated neutrophils (TANs) toward the terminal pro‐tumor subset (TAN‐1), thereby mediating immune evasion [34].

The aforementioned mechanism of glucose metabolism regulating immunosuppressive cells indicates that targeting glucose metabolism to inhibit immune cell function represents a novel therapeutic strategy for reversing immunosuppression and enhancing antitumor immune cells. Combining TGF‐β signaling pathway inhibitors (SB‐431542) with PKM2 activity inhibitors (thymol) disrupts the TGF‐β‐PKM2/p‐STAT glycolytic axis, downregulates PD‐L1 expression, reverses immune suppression, and inhibits tumor growth, offering a promising combination therapy for bladder cancer [28]. Zhou et al. [31] found that the CXCR2 antagonist (SB225002) reduces PMN‐MDSC aggregation, enhances CD8^+^T cell infiltration, and potentiates anti‐PD‐1 efficacy. Additionally, in breast cancer, targeting the protein C–E‐cad encoded by circular RNA was found to reduce PMN‐MDSC numbers, restore T cell function, and synergistically inhibit tumor growth with PD‐1 blockade [32]. Similarly, Eleutheroside A (EA) attenuates the immunosuppressive capacity of MDSCs in gastric cancer by inhibiting the PI3K/AKT/mTOR pathway and HIF1α‐mediated glycolysis, offering a novel strategy for reshaping the immunosuppressive microenvironment [35]. Furthermore, studies have demonstrated that IL‐37 can also alleviate MDSC‐mediated immunosuppression through metabolic reprogramming. This occurs because IL‐37 enhances glycolysis and oxidative phosphorylation in MDSCs, increasing ATP release and thereby reducing their inhibitory effects on T cells [36]. Recent findings reveal that depletion of the ubiquitin ligase UBE2I can reprogram glycolytic metabolism to promote pro‐inflammatory activated TAM polarization and enhance the efficacy of anti‐PD‐L1 therapy in ovarian cancer [37]. These discoveries unveil novel immunotherapeutic approaches targeting the metabolism of immunosuppressive cells.

The sustained function of immunosuppressive cells also depends on their efficient uptake and utilization of lactate within the microenvironment. For instance, in highly glycolytic tumor microenvironments, Tregs actively take up lactate via MCT1, which induces NFAT1 nuclear translocation and selectively upregulates PD‐1 expression, whereas PD‐1 expression in effector T cells is suppressed [38]. Additionally, upregulation of lactate dehydrogenase A and lactate transporters also promotes immune‐suppressive cell functions. Hepatocellular carcinoma cells lacking ACVR2A upregulate LDHA and MCT4, increasing lactate secretion and promoting Treg aggregation, thereby driving resistance to PD‐1 inhibitors [39]. In gliomas, high expression of LDHA and MCT1 leads to lactate accumulation, which activates the tumor‐associated macrophage surface receptor GPR65 and its downstream cAMP/PKA/CREB signaling cascade. This triggers mitochondrial dysfunction and the release of high‐molecular‐weight nucleoprotein 1 (HMGB1). These events drive malignant progression and reinforce the alternative activation‐type TAM polarization [40]. Similarly, LKB1 loss‐of‐function mutations upregulate MCT4 and enhance lactate secretion. Extracellular lactate mediates primary resistance to PD‐1 blockade by binding to GPR81 receptors on immune cell surfaces, promoting alternative activation‐type TAM polarization and inducing T cell exhaustion. In LKB1‐mutant lung adenocarcinoma, silencing MCT4 or blocking GPR81 reverses immunosuppression, restores antitumor immune function, and overcomes immunotherapy resistance [41]. Lactic acid also catalyzes the lysine‐72 lactylation modification of the cytoskeletal linker protein MOESIN, enhancing its interaction with TGF‐β receptor I. This activates the SMAD3 pathway to promote Treg stability and immunosuppressive function [42]. Therefore, MOESIN lactylation in Tregs can serve as a biomarker for predicting immunotherapy responsiveness. Additionally, lactate can recruit CCR2+ PMN‐MDSCs to the tumor microenvironment by activating the colorectal cancer cell surface receptor HCAR1 to upregulate CCL2/CCL7 expression, while simultaneously suppressing CD8^+^T cell function, ultimately driving immune escape [43]. On the other hand, CAFs can utilize lactate produced by tumor metabolism as an alternative energy source. Lactate metabolism not only promotes CAF proliferation but also upregulates IL‐6 expression, thereby suppressing cytotoxic immune cell function and reinforcing the immunosuppressive tumor microenvironment [44]. Thus, lactic acid and IL‐6 jointly form the dual foundation for maintaining CAF metabolic activity and regulatory function. IL‐6 enhances glucose uptake by cancer cells and competes for glucose supply to immune cells, thereby increasing HIF1α expression and inducing tumor immune suppression [45]. Therefore, anti‐IL‐6 receptor antibodies can be used to overcome tumor‐induced immunosuppression and improve survival rates in patients with various cancers.

Recent advances in nanotechnology have enabled precise targeted intervention in glucose and lactate metabolism, thereby reversing the activation of immunosuppressive cells. Tang et al. [46] designed a self‐oxidizing nanoplatform (ML@Lip) by co‐encapsulating lactate oxidase and manganese porphyrin within liposomes. This platform consumes lactate, promotes pro‐inflammatory TAM polarization, and remodels the immune microenvironment. Additionally, manganese porphyrin catalyzes lactate decomposition to produce hydrogen peroxide and subsequent oxygen, thereby enhancing sonodynamic therapy (SDT)‐induced immunity. Long et al. [47] designed MPDA nanoparticles loaded with violaxanthin and modified with hyaluronic acid (SHKHA‐MPDA). These nanoparticles suppress PKM2 expression and glycolysis in tumor cells, thereby reducing lactate metabolic flux. This leads to decreased MDSC numbers, increased CD8^+^T cell counts, activated dendritic cells, and a remodeled tumor immune microenvironment. Additionally, the intelligent nanomissile HAAT‐Pd developed by the Gao team [48] induces tumor cell energy depletion and reduces lactate production by dual inhibition of energy metabolism (oxidative phosphorylation + glycolysis). Simultaneously, it reverses CAF activation and epithelial‐mesenchymal transition (EMT) processes by suppressing the TGF‐β/Smad signaling pathway. Furthermore, HAAT‐Pd synergizes with photothermal therapy to induce immune cell apoptosis, ultimately reshaping the immunosuppressive microenvironment into an immune‐activated state, effectively inhibiting tumor progression and metastasis. Recent research has discovered [49] that using extracellular vesicles (cEVs) derived from cancer‐associated fibroblasts (CAFs) as carriers enables targeted delivery of BAY‐876 to GLUT1‐high‐expressing CAFs and tumor cells. cEV‐BAY‐876 (cEVB6) therapy significantly generates a glucose‐rich, low‐lactic acid tumor microenvironment, reverses the activated CAF phenotype, enhances cytotoxic T lymphocyte infiltration, and thereby improves the efficacy of anti‐cancer immunotherapy.

Glucose metabolism pathways primarily promote immune cell escape through low glucose levels, high lactate levels, and the regulation of metabolic enzyme and transporter expression. Consequently, glucose metabolism represents a critical therapeutic target in cancer treatment. Extensive research demonstrates that combining drugs targeting glucose metabolism with immune checkpoint inhibitors significantly enhances immunotherapy efficacy. Additionally, drug delivery systems (such as nanotechnology) enable precision treatment. Currently, clinical trials of drugs targeting glucose metabolic enzymes and transporters remain in preclinical research and early clinical exploration phases (Table 1), with insufficient clinical trials. This may stem from challenges in drug development due to the structural complexity of targets, narrow therapeutic windows, and the presence of compensatory mechanisms, all contributing to difficulties in applying glucose metabolism‐targeting drugs. Additionally, the toxicity associated with prolonged use of nanotechnology or drug delivery systems, along with their delivery rates and selectivity within tumor tissues, pose significant challenges for clinical translation. Therefore, future efforts should focus on multidisciplinary research to thoroughly elucidate the mechanisms underlying glucose metabolism and immune interactions, explore combination therapies, and develop more precise and safer treatment strategies to enhance the efficacy of tumor immunotherapy and improve patient survival rates.

**TABLE 1:** Clinical trials of glucose metabolism drugs in cancer treatment.

Target		Drug	Mechanism	Targeted immune cells	Cancer Types	Clinical Trial	Combination therapy
Transporter protein	SGLT‐2	Canagliflozin	Inhibiting Sodium‐Glucose Cotransporter 2 (SGLT2) function and indirectly modulates PD‐L1 expression and function	CD8+T	Colorectal Cancer	NCT07076823 (I) Recruiting	None
		Dapagliflozin Dapagliflozin			Pancreas Cancer	NCT04542291 (I) Completed	None
			Directly inhibiting SGLT2 on tumor cells and suppresses insulin/IGF‐1 signaling pathways.		Prostate Cancer	NCT04887935(I) Recruiting	None
	MCT4	AZD3965	Inhibiting lactate efflux induces Intracellular acidosis in tumor cells	Tregs	Adult Solid Tumor, Diffuse Large B Cell Lymphoma, Burkitt Lymphoma,	NCT01791595 (I) Completed	Monotherapy
key enzyme	GAPDH	Dimethyl fumarate	Inhibits GAPDH activity, downregulating aerobic glycolysis levels in tumor cells	CD8+T, NK	Glioblastoma Multiforme	NCT02337426 (I) Completed	Temozolomide, Radiation Therapy
					Cutaneous T Cell Lymphoma	NCT02546440 (II) Completed	None

*Note*: Retrieved from https://clinicaltrials.gov/.

## 3 Regulatory Mechanisms and Therapeutic Strategies of Amino Acid Metabolism on Immune Cells

**FIGURE 3:**
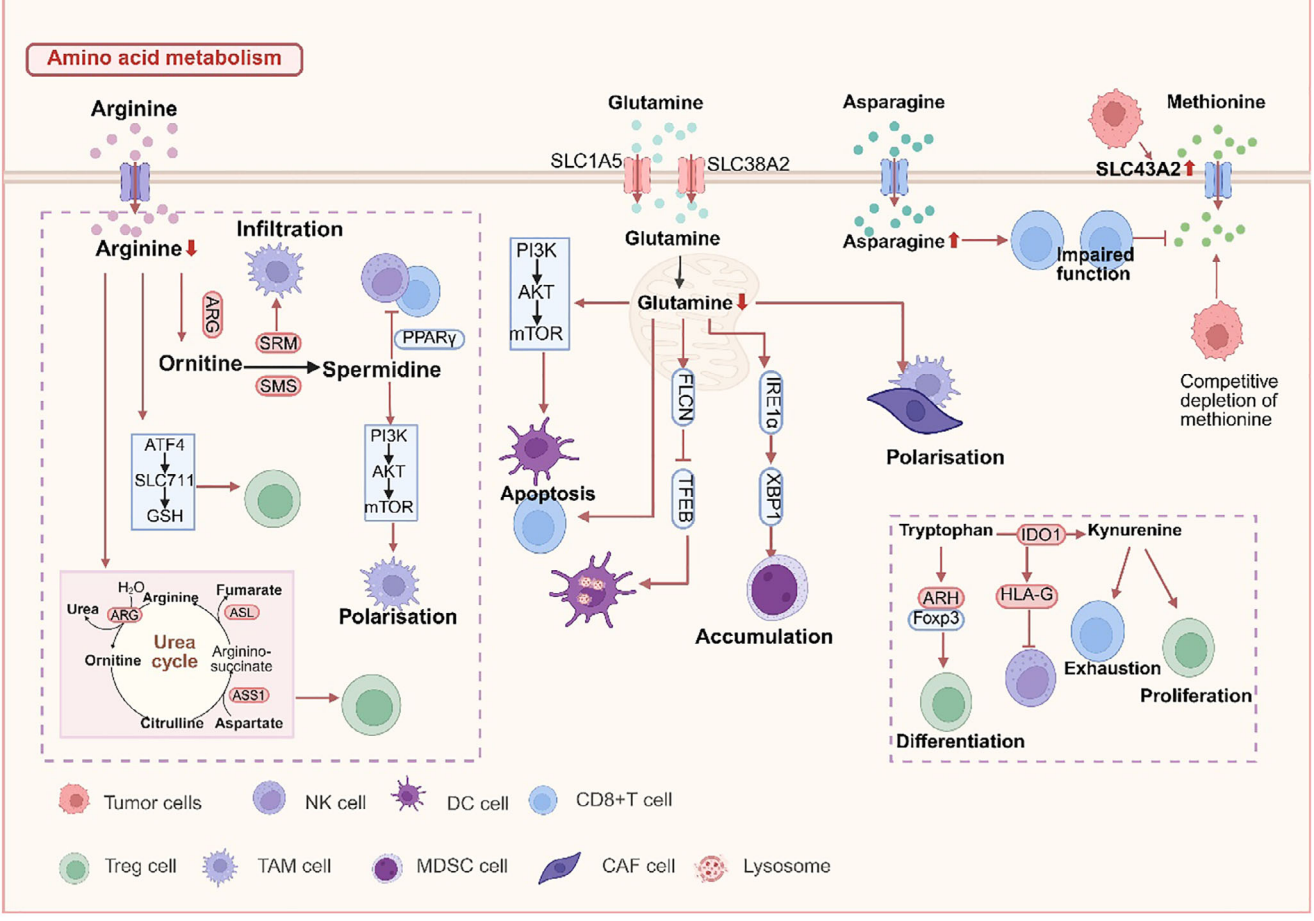
Regulatory Mechanisms of Amino Acid Metabolism on Immune Cells. Abnormalities in amino acid metabolism pathways—including glutamine, asparagine, methionine, tryptophan‐kynurenine, arginine, urea cycle, and polyamine synthesis—are closely associated with immune cell regulation. Glutamine induces apoptosis in antitumor immune cells via signaling pathways such as PI3K/AKT and FLCN/TFEB, while promoting the accumulation and polarization of immune‐suppressive cells through pathways like IRE1α/XBP1. Excessive asparagine intake impairs CD8^+^T cell function. Conversely, tumor cells competitively deprive CD8^+^T cells of methionine, weakening their antitumor immune function. The tryptophan‐kynurenine pathway, via IDO1 and AHR, exhausts CD8^+^T and NK cells while promoting Treg differentiation and expansion. The metabolic pathways of arginine, the urea cycle, and polyamine synthesis are intricately interconnected. Low arginine concentrations, overexpressed metabolic enzymes, and spermine can suppress anti‐tumor immune cell activity through PI3K/AKT, ATF4/SLC711/GSH, and other pathways, promoting the infiltration and polarization of immunosuppressive cells. Abbreviations: IDO1: Indoleamine 2,3‐dioxygenase 1; AHR: Aryl Hydrocarbon Receptor; ARG1: Arginase 1; SRM: Spermidine Synthase; SMS: Spermine Synthase; ASL: Argininosuccinate Lyase; ASS1: Argininosuccinate Synthase 1. Created in https://BioRender.com.

Amino acid metabolism, including glutamine metabolism, serine metabolism, arginine metabolism, and tryptophan‐kynurenine metabolism, participates in numerous immune cell pathways and determines immune cell function [50, 51]. Metabolic competition between tumor cells and immune cells also occurs in the realm of amino acid metabolism. Tumor cells preferentially uptake glutamine, serine, and arginine, leading to depletion of these nutrients in the TME. This nutrient deprivation inhibits the proliferation and activation of anti‐tumor immune cells while promoting the function of immune‐suppressive cells. Consequently, key regulators of this pathway can be considered potential therapeutic targets (Figure 3).

### 3.1 Regulatory Mechanisms and Therapeutic Strategies of Amino Acid Metabolism in Counteracting Anti‐Tumor Immune Cells

Glutamine is one of the essential amino acids, and alterations in its metabolism profoundly influence the fate of anti‐tumor immune cells. For instance, glutamine deprivation induces mitochondrial damage and apoptosis in CD8^+^T cells, impairing the functional capacity of CD8^+^T cell infiltration in hepatocellular carcinoma [52]. Classical dendritic cells (cDCs) comprise two distinct subpopulations: cDC1 and cDC2. The cDC1 subpopulation plays a critical role in antigen cross‐presentation and tumor immunity, with its function regulated by glutamine levels. Reduced glutamine levels impair cDC1 cell function by modulating the mTORC1 signaling pathway [53]. Furthermore, the expression of the glutamine transporter SLC38A2 is also closely associated with antitumor immune cell responses. Research indicates that single‐cell RNA sequencing reveals X‐box binding protein 1 (XBP1) can directly suppress SLC38A2 expression, thereby reducing intracellular glutamine levels and leading to CD8^+^T cell immune dysfunction [54]. Tumor cells competitively deplete glutamine via the SLC38A2 transporter and suppress the FLCN‐TFEB signaling pathway in cDC1 cells, thereby disrupting cDC1 function and inducing immune escape. Intratumoral glutamine supplementation or modulation of the SLC38A2/FLCN pathway can remodel cDC1 function and overcome tumor‐mediated immunosuppression [55]. Therefore, effectively regulating glutamine metabolism is a crucial pathway for enhancing the function of antitumor immune cells.

Given the pivotal role of glutamine metabolism in antitumor immune cell‐mediated immune responses, inhibiting glutamine and glutaminase (GLS) utilization in tumor cells has emerged as a promising therapeutic strategy to enhance immunotherapy efficacy. For example, the glutamine antagonist DRP‐104 reverses T cell exhaustion by blocking glutamine‐dependent nucleotide synthesis, enhances CD4^+^and CD8^+^T cell function, reduces Treg numbers, and improves responsiveness to anti‐PD‐1 therapy [56]. Similarly, the glutamine antagonist prodrug JHU083 comprehensively inhibits glutamine‐dependent metabolic pathways in tumor cells, reduces HIF1α and phosphorylated c‐MYC levels, induces tumor cell apoptosis, and promotes the acquisition of a stem cell‐like phenotype in CD8^+^T cells to enhance antitumor immunity [57]. The glutamine transporter inhibitor V‐9302 selectively blocks glutamine uptake in tumor cells but not T cells. T cells compensate by upregulating alternative glutamine transporters, thereby enhancing Th1 and CD8^+^T cell activation, strengthening antitumor immunity, and simultaneously inhibiting tumor cell metabolism [58]. Research on another glutamine transporter, SLC25A22, has also confirmed that this targeting strategy enhances the cytotoxicity of CD8^+^T cells and increases tumor sensitivity to anti‐PD‐1 therapy [59]. Yan et al. [60] developed a manganese‐coordination‐driven dual‐targeted nanomedicine (HA/E‐M@corydin nanoparticle) that simultaneously modulates glutamine metabolism and cancer stem cell properties. This nanomedicine alleviates immunosuppressive stress through a triple mechanism: inhibiting glutamine metabolism to elevate extracellular glutamine concentration, enhancing cGAS‐STING signaling to promote dendritic cell maturation, and concurrently diminishing cancer stemness. In summary, targeting glutamine metabolism in tumor cells represents a highly promising strategy for enhancing immunotherapy efficacy.

Additionally, other amino acid metabolic dysregulations—including asparagine, methionine, serine, and tryptophan‐kynurenine—simultaneously promote immune escape mediated by antitumor immune cells. Unlike glutamine, asparagine exerts a stage‐dependent effect on CD8^+^T cells. During the early activation phase, elevated asparagine levels enhance CD8^+^T cell activation and effector function by binding to the Src family tyrosine kinase LCK and modulating its phosphorylation at Tyr394 and Tyr505 sites, thereby amplifying T cell receptor (TCR) signaling [61]. Conversely, during the late differentiation stage, asparagine promotes immune evasion by inhibiting type I interferon signaling in bladder cancer [62]. However, restricting asparagine intake enhances CD8^+^T cell proliferation and effector potential by activating NRF2—this mechanism reduces overall glucose and glutamine consumption, elevates intracellular nucleotide levels, and thereby reprograms metabolic pathways [63]. Therefore, restricting asparagine intake may serve as a clinically viable strategy to enhance the efficacy of cancer immunotherapy. Tumor cells also exploit methionine metabolism to achieve immune evasion. By overexpressing the methionine transporter SLC43A2, tumor cells competitively deplete methionine in the tumor microenvironment, thereby limiting CD8^+^T cell uptake of this substance and impairing their antitumor function [64]. Serine metabolism also regulates the activation of anti‐tumor immune cells. Targeting serine synthesis can prevent the exhaustion of anti‐tumor immune cells. For example, the phosphoglycerate dehydrogenase inhibitor NCT503 significantly enhances NK cell activation by inhibiting the FOS/FOSL2 signaling pathway, thereby improving the efficacy of anti‐PD‐1 immunotherapy [65]. The key enzyme in the tryptophan‐kynurenine pathway, indoleamine 2,3‐dioxygenase 1 (IDO1), is induced by cytokines such as interferon‐γ (IFN‐γ) in the tumor microenvironment. Upregulation of IDO1 increases HLA‐G expression, thereby suppressing NK cell‐mediated antitumor immunity and promoting tumor immune escape [66].

The biosynthesis of polyamines primarily utilizes ornithine and arginine as precursors, ultimately yielding putamine, spermidine, spermine, and functional molecules. Complex interactions exist between polyamine metabolism and immune cells. Targeting key points in polyamine metabolism represents a promising strategy to disrupt tumor cell metabolic dependencies and modulate the immune tumor microenvironment (TME) to enhance antitumor immunity. Spermine can drive tumor progression by diminishing CD8^+^T cell numbers and cytotoxic function. However, knocking down ornithine decarboxylase—the rate‐limiting enzyme in spermine synthesis—significantly prolonged animal survival despite having no effect on cancer cell growth in vitro [67]. Research has revealed that during the progression of colon cancer, energy metabolism disorders in tumor cells drive them to plunder polyamines from NK cells. This leads to suppression of c‐Myc in NK cells, impairing their glycolytic function and consequently reducing their cytotoxic activity. Conversely, administration of specific spermidine reverses c‐Myc inhibition and glycolytic energy supply disruption in NK cells, restoring their cytotoxic activity [68]. Spermine can also downregulate cellular membrane cholesterol levels, thereby specifically inhibiting T cell receptor (TCR) signaling. Validation in a homologous mouse model revealed that spermine is abundantly present in the tumor immune microenvironment, and administration of polyamine synthesis inhibitors significantly enhanced CD8^+^T cell‐dependent antitumor responses [69]. The polyamine biosynthesis inhibitor difluoromethylornithine (DFMO) has also demonstrated promising clinical potential. Combining DFMO with PD‐1 blockade significantly enhances the survival and activation of intratumoral CD8^+^T cells, thereby improving treatment efficacy [70]. Thus, co‐administering polyamine synthesis inhibitors with immune checkpoint antibodies can induce a more potent antitumor immune response.

### 3.2 Regulatory Mechanisms and Therapeutic Strategies of Amino Acid Metabolism in Immunosuppressive Cells

Glutamine competition within the tumor microenvironment also drives immunosuppressive reprogramming. Tumor cells and myeloid cells compete for glutamine uptake via the transporter SLC1A5, disrupting endoplasmic reticulum homeostasis and inducing the unfolded protein response (UPR). This process activates the IRE1α/XBP1 pathway, upregulates GPR109A expression in myeloid cells, and promotes immune evasion. Blocking GPR109A reduces G‐MDSC and alternative‐activated TAM numbers while restoring CD8^+^T cell‐mediated antitumor immunity in hepatocellular carcinoma [71]. Furthermore, glutathione peroxidase 4 (GPX4) expression positively correlates with infiltrating alternative‐activated macrophages. Silencing GPX4 enhances lipid peroxidation, reduces ornithine accumulation, and reprograms tumor‐associated macrophages toward an anti‐tumor phenotype [72]. CAFs are one of the primary cell types in tumors that highly express glutamine synthetase. Under glutamine‐restricted conditions, CAFs can produce and secrete glutamine, providing energy for tumor bioenergetic metabolism and biosynthesis [73]. Tumor‐derived palmitic acid activates the TLR4‐Syk‐NF‐κB cascade in CAFs, inducing inflammatory CAF polarization and IL‐6‐mediated glutamine synthesis, thereby promoting TAM polarization [74]. Therefore, targeting glutamine synthesis in CAFs may represent a viable strategy for reprogramming the immunosuppressive tumor microenvironment.

The tryptophan‐kynurenine (Kyn) metabolic pathway regulates immune responses in inflammatory and immunosuppressive cells. Tryptophan deprivation induces AHR overexpression via a GCN2‐dependent mechanism and enhances SLC7A5 (LAT1) expression, increasing intracellular kynurenine uptake, sensitizing the AHR pathway, and ultimately promoting Treg differentiation [75]. Tryptophan can also directly activate AHR nuclear translocation, enhancing its binding to the Foxp3 promoter, thereby upregulating Foxp3 expression and promoting Treg differentiation [76]. In nasopharyngeal carcinoma, FLI1 recruits CBP and amplifies IFN‐γ‐induced chromatin accessibility, thereby enhancing IDO1 transcription and kynanogenesis, which subsequently drives CD8^+^T cell exhaustion and Treg expansion [77]. Inhibiting IDO activity effectively blocks the tryptophan‐AHR axis, reduces Treg infiltration, and enhances CD8^+^T cell immunity, offering a novel therapeutic approach for colitis‐associated colorectal cancer. Clinical trials of IDO1 small‐molecule inhibitors are currently underway (Table 2), revealing the ability of IDO1 inhibitors to modulate the immune system.

**TABLE 2:** Clinical trials of amino acid metabolism drugs in cancer treatment.

Target		Drug	Mechanism	Targeted immune cells	Cancer Types	Clinical Trial	Combination therapy
Amino acid		ADI‐PEG20	Catalyzes the breakdown of arginine in the blood into citrulline, rapidly depleting exogenous arginine.	NK, CD8+T	Prostate Cancer	NCT06085729(I/II) Recruiting	Carboplatin and Cabazitaxel
					Non‐small Cell Lung Cancer	NCT05616624(I/II) Active, not recruiting	Gemcitabine and Docetaxel
					Advanced Solid Cancers	NCT03254732 (I) Terminated	Pembrolizumab
					Metastatic Melanoma, Skin Cancer	NCT00520299 (I/II) Completed	Transarterial chemoembolization
					Unresectable Hepatocellular Carcinoma	NCT02006030 (II) Completed	None
					HER2 Negative Metastatic Breast Cancer	NCT01948843 (I) Completed	Doxorubicin
					Advanced Pancreatic Cancer	NCT02101580 (I) Completed	Nab‐Paclitaxel and Gemcitabine
					Soft Tissue Sarcoma	NCT05712694 (III) Recruiting	None
					With High Arginine Level and Unresectable Hepatocellular Carcinoma	NCT05317819 (III) Recruiting	None
					Uveal Melanoma	NCT03922880 (I) Completed	nivolumab and ipilimumab
					Hepatocellular Carcinoma	NCT06034977 (II) Recruiting	Lenvatinib
Amino acid metabolism enzyme	GLS1	CB‐839 (telaglenastat)	Selectively inhibiting glutaminase 1 (GLS1) blocks the glutamine metabolic pathway.	CD8^+^ T	Colon Cancer, Colorectal Cancer, Rectal Cancer, Solid Tumor	NCT02861300 (I/II) Completed	Capecitabine
					Solid Tumor,Clear Cell Renal Cell Carcinoma, Triple‐Negative Breast Cancer, Colorectal Cancer, Renal Cell Carcinoma	NCT03875313 (I/II) Completed	Talazoparib
					Triple Negative Breast Cancer	NCT03057600 (II) Completed	Paclitaxel
					Advanced Renal Cell Carcinoma, Metastatic Renal Cell Carcinoma	NCT03428217 (II) Completed	Cabozantinib
					Clear Cell Renal Cell Carcinoma, Melanoma, Non‐small Cell Lung Cancer	NCT02771626 (I/II) Completed	Nivolumab
					Advanced Myelodysplastic Syndrome	NCT03047993 (I/II) Completed	Azacitidine
	IDO1	Epacadostat (INCB024360)	Inhibiting IDO1 reduces kynurenine production.	Treg, MDSC	Squamous Cell Carcinoma of Head and Neck, Non‐Small Cell Lung Cancer, Glioblastoma, Melanoma, Diffuse Large B‐Cell Lymphoma, Colorectal Cancer, Ovarian Cancer	NCT02327078 (I/II) Completed	Nivolumab
					Platinum‐resistant Ovarian Cancer, Platinum‐resistant Fallopian Cancer, Platinum‐resistant Peritoneal Cancer	NCT02575807 (I/II) Terminated	CRS‐207
					Melanoma	NCT01961115 (II) Completed	Vaccine therapy
					Glioma	NCT03532295 (II) Active, not Recruiting	Retifanlimab, Radiation, Therapy, bevacizumab
					Gastrointestinal Stromal Tumors	NCT03291054 (II) Terminated	Pembrolizumab
					Gastric Adenocarcinoma, Gastroesophageal Junction Adenocarcinoma	NCT03196232 (II) Terminated	Pembrolizumab
					Urothelial Cancer	NCT03361865 (III) Terminated	Pembrolizumab
					Melanoma	NCT02752074 (III) Completed	Pembrolizumab
	ARG1	CB‐1158 (INCB001158)	Inhibiting arginase restores T cell function.	TAM, MDSC, CD8^+^ T	Solid Tumors	NCT03361228 (I/II) Terminated	Epacadostat, Pembrolizumab
					Colorectal Cancer, Gastric Cancer, Head and Neck Cancer, Lung Cancer, Renal Cell Carcinoma, Bladder Cancer, Urothelial Cancer, Mesothelioma	NCT02903914 (I) Completed	Pembrolizumab
					Relapsed or Refractory Multiple Myeloma	NCT03837509 (I/II) Completed	Daratumumab SC
					Biliary Tract Cancer, Colorectal Cancer, Endometrial Cancer, Gastroesophageal Cancer, Ovarian Cancer	NCT03314935 (I/II) Completed	Chemotherapy
	ARG1, ARG2	OATD‐02	Simultaneously inhibit ARG1 and ARG2 to restore arginine levels.	T cell	Colorectal Cancer, Ovarian Cancer, Pancreatic Cancer or Renal Cell Carcinoma	NCT05759923 (I) Recruiting	None

*Note*: Retrieved from https://clinicaltrials.gov/.

Abnormal arginine metabolism within tumors promotes the reprogramming of immune‐suppressive cells. Low concentrations of arginine (∼20 µM) activate the ATF4‐SLC7A11‐GSH metabolic axis, reprogramming activated T cells into a Treg‐like inhibitory phenotype, thereby facilitating tumor immune escape [78]. Additionally, the IL‐4/IL‐13 signaling pathway induces macrophage expression of cholesterol 25‐hydroxylase (CH25H) by activating STAT6, thereby generating 25‐hydroxy cholesterol (25HC). This metabolite inhibits mTORC1 and activates AMPKα by binding to the GPR155 receptor, thereby enhancing STAT6 phosphorylation and upregulating arginase 1 (ARG1) expression, which further strengthens the immunosuppressive metabolism of alternatively activated macrophages [79]. The accumulation of acetate in the tumor microenvironment activates the Gαq/Ca^2^^+^/PPAR‐γ pathway via the FFAR2 receptor on myeloid‐derived suppressor cells, upregulating ARG1 expression and depleting L‐arginine, thereby suppressing CD8^+^T cell activity. Inhibition of FFAR2 or combined treatment with L‐arginine supplementation and PPAR‐γ antagonists reverses MDSC‐mediated T cell dysfunction and enhances checkpoint blockade efficacy [80]. Moreover, inhibiting the ARG pathway can overcome tumor‐associated macrophage‐mediated immune suppression.

The urea cycle (UC) is a critical pathway in amino acid metabolism for managing ammonia toxicity, closely intertwined with the metabolism of arginine. Tumor cells rely on the reprogramming of the urea cycle to enhance ammonia utilization and processing, thereby promoting their own growth and proliferation while driving immune suppression [81]. ARG is also a key hydrolytic enzyme in the urea cycle, catalyzing the hydrolysis of L‐arginine to produce urea and L‐ornithine [82]. Recent research reveals that Tregs detoxify ammonia via the urea cycle by upregulating argininosuccinate lyase (ASL). Simultaneously, ammonia is converted into spermine through spermine synthase, regulated by the FOXP3 transcription factor. Spermine directly interacts with PPARγ, thereby comprehensively regulating the transcription of multiple mitochondrial complex proteins. This enhances oxidative phosphorylation and promotes the immunosuppressive function of Tregs. Clinically, it has been observed that dying tumor cells undergoing anti‐PD‐1 therapy release ammonia through transamidation, which amplifies Treg function and leads to resistance to immunotherapy [83]. Targeting amino acid metabolism to inhibit Tregs offers a potential strategy for anti‐tumor immunotherapy. Zhao et al. [84] revealed that AQP5+ gastric cancer stem cells suppress NK cell activity by limiting nitric oxide synthesis through the establishment of a low‐arginine microenvironment. AQP5 downregulates arginine succinate synthase 1 (ASS1) by inhibiting DHX9 nuclear translocation, while simultaneously stabilizing glutamate‐glutamine ligase (GLUL). This redirects urea cycle activity toward the tricarboxylic acid cycle, storing conserved nitrogen as glutamine. Combined use of ULK1 inhibitors with L‐arginine synergistically inhibits AQP5+ tumor progression, offering novel therapeutic insights.

Given that ARG plays a pivotal role in tumor growth and immune evasion through multiple mechanisms, it has emerged as a promising therapeutic target for cancer treatment. OATD‐02 is a novel dual arginase (ARG1/ARG2) inhibitor that reprograms tumor metabolism by restoring L‐arginine availability and reducing polyamine levels, thereby shifting the tumor microenvironment (TME) toward a more immunostimulatory state. OATD‐02 is currently undergoing a Phase I/II clinical trial (NCT05759923), which will further elucidate its safety and therapeutic efficacy [85]. Additionally, small‐molecule compounds or vaccines targeting ARG have been developed and advanced to the clinical trial stage (Table 1). Nanotechnology also serves as a means to inhibit tumor growth and achieve precision therapy. A nanoplatform composed of tumor‐derived particles, loaded with paeoniflorin‐3′‐O‐glucoside and indocyanine green (PG/ICG@MPs), can precisely target tumor sites, suppress ARG activity, and activate nitric oxide synthase. This dual regulation reduces the number of alternative‐activated TAMs while promoting pro‐inflammatory activated TAM polarization, thereby reshaping the tumor microenvironment into an immunologically active state [86]. Yan et al. [87] found that ginseng‐derived nanoparticles (GDNPs) reprogram tumor‐infiltrating macrophages by reducing ARG1 production through modulation of the mTOR‐T‐bet axis, thereby improving T cell exhaustion and enhancing CD8^+^T cell proliferation. These findings provide new insights into the mechanisms of suppressing immune cell function and alleviating antitumor immune cell exhaustion. This may facilitate the development of innovative therapeutic strategies in the field of cancer treatment.

Polyamine metabolism also plays a crucial role in regulating immune‐suppressive cells. Polyamines derived from arginine metabolism enhance pro‐tumorigenic TAM polarization through thymidine DNA glycosyltransferase (TDG)‐mediated DNA demethylation [88]. The arginine‐polyamine‐TDG axis targeting cancer cells and macrophages significantly inhibits breast cancer growth, highlighting its therapeutic potential. Spermidine synthase (SRM) expression positively correlates with TAM infiltration and hepatocellular carcinoma progression. SRM promotes hepatocellular carcinoma progression via a paracrine mechanism by altering the immunometabolic state of macrophages [89]. Additionally, spermine synthase (SMS) is significantly upregulated in tumor cells, with its expression levels positively correlated with the immunosuppressive microenvironment and predictive of poor prognosis in hepatocellular carcinoma patients. However, elevated SMS levels in HCC cells primarily mediate immune evasion through its metabolite spermine. Spermine reprograms TAMs by activating the PI3K‐Akt‐mTOR‐S6K signaling pathway, inducing TAMs to adopt an alternative activated polarization. This leads to diminished antitumor function in CD8^+^T cells [69]. Zhang et al. [90] proposed an immunotherapy strategy leveraging the synergistic effects of polyamine metabolism‐induced aldehyde production and oxidative stress. This approach primarily induces mitochondrial dysfunction, DNA damage, and lipid peroxidation accumulation by overproducing hydrogen peroxide and acrolein, thereby inducing oxidative/carbonyl stress while suppressing endogenous antioxidant systems. Additionally, these nanoparticles enhance the recruitment of mature dendritic cells and T cells within the tumor microenvironment (TME), thereby suppressing lung metastasis and tumor recurrence. Additionally, Hai et al. [91] developed an innovative tumor‐specific nanocarrier enabling synergistic delivery of aPD‐1 and MDK siRNA. This approach significantly suppressed alternative activation polarization and polyamine metabolism in TAMs and MDSCs, thereby overcoming the immunosuppressive tumor microenvironment. It achieved remarkable therapeutic efficacy with minimal side effects in hepatocellular carcinoma treatment.

As reviewed, amino acid metabolism plays a crucial role in regulating immune suppression. In recent years, therapeutic strategies targeting amino acid metabolism have also emerged as a research hotspot in the field of oncology. However, most of these approaches focus on inhibitors of amino acids and amino acid metabolic enzymes, many of which have already entered clinical trial phases (Table 2). However, inhibitors targeting transporters have received limited attention in both preclinical and clinical studies. Although research has confirmed that transporters (such as SLC38A2 and SLC43A2) regulate immune cell function through amino acid metabolism, preclinical studies have encountered failures in clinical translation due to the activation of compensatory pathways. Furthermore, numerous studies have demonstrated that nanomedicines enable precise delivery of amino acid metabolism drugs, enhancing drug accumulation in tumor tissues. Although therapeutic strategies targeting amino acid metabolism hold broad application prospects, clinical translation still faces challenges including drug toxicity, resistance, tumor suppression, and individual variability, necessitating further exploration and research.

## 4 Regulatory Mechanisms and Therapeutic Strategies of Lipid Metabolism on Immune Cells

**FIGURE 4:**
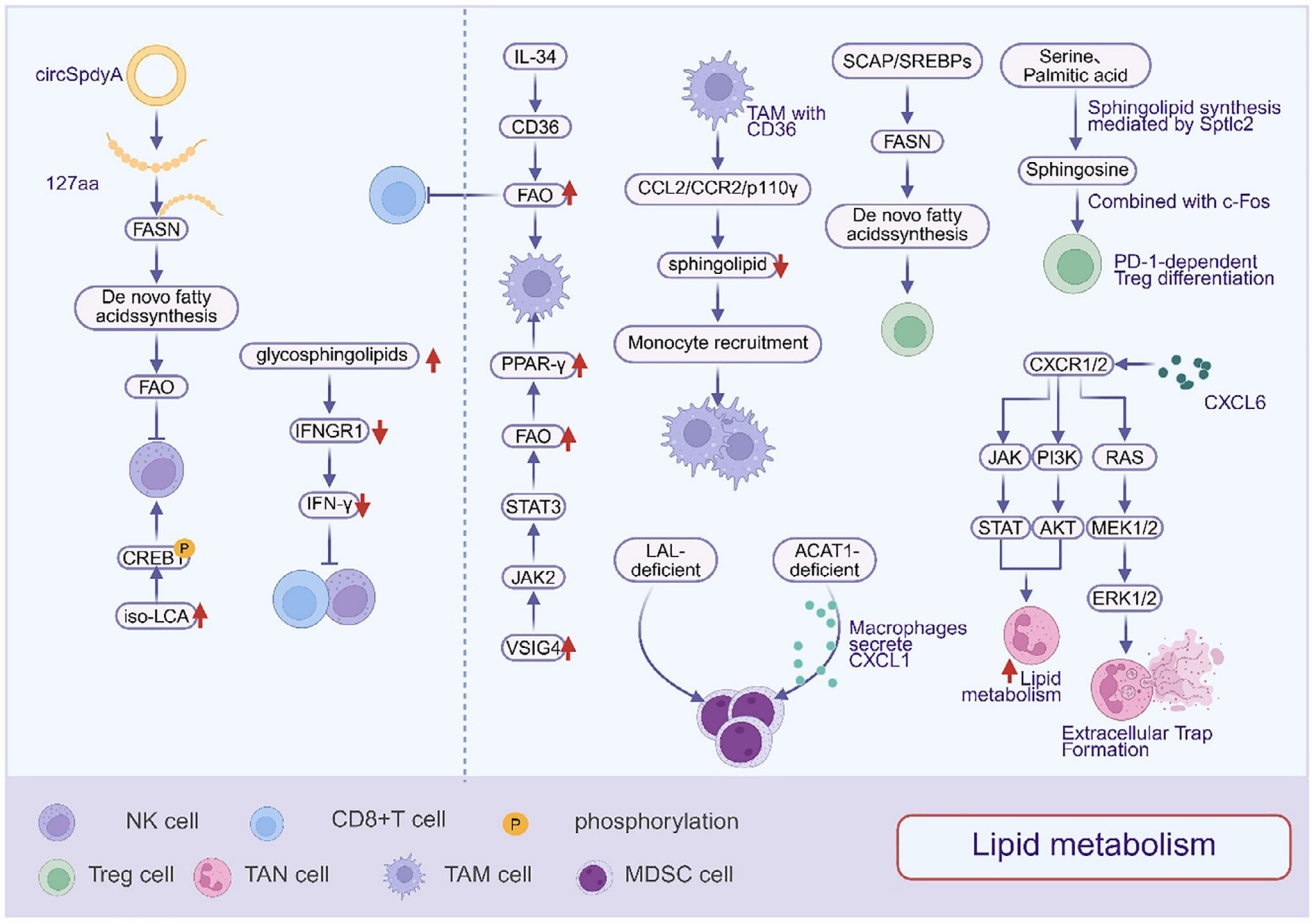
Regulatory Mechanisms of Lipid Metabolism on Immune Cells. The accumulation of metabolites during lipid metabolism (such as glycosphingolipids, lipids, and iso‐LCA), activation of the metabolic enzyme FASN, and enhanced FAO collectively suppress the activity of antitumor immune cells while promoting the immunosuppressive functions of immune‐suppressive cells. Abbreviations: FASN: Fatty Acid Synthase; FAO: Fatty acid oxidation; iso‐LCA: isolithocholic acid. Created in https://BioRender.com.

Lipid metabolism profoundly influences immune cell activation, cytotoxicity, and immune surveillance by regulating de novo sphingolipid synthesis, fatty acid metabolism, cholesterol homeostasis, and lipid peroxidation‐mediated oxidative stress. Understanding lipid metabolic characteristics within the TME not only reveals intrinsic links between immune cell function and metabolic reprogramming but also offers novel perspectives for overcoming current immunotherapy bottlenecks (Figure 4).

### 4.1 Regulatory Mechanisms and Therapeutic Strategies of Lipid Metabolism in Counteracting Anti‐Tumor Immune Cells

Tumor cells can promote immune resistance by activating the de novo sphingolipid synthesis pathway, leading to glycosphingolipid accumulation. This downregulates the membrane expression of interferon‐γ receptor 1 (IFNGR1), weakens IFN‐γ signaling, and suppresses immune surveillance by NK cells and CD8^+^T cells [92]. Additionally, gastric cancer stem cells promote cholesterol metabolism by upregulating the transcription factor sterol regulatory element‐binding protein 2 (SREBP2), thereby altering membrane composition and enhancing tumor cells' tolerance to NK cell‐mediated killing activity [93].Additionally, enzymes and metabolites involved in lipid metabolism also suppress the activity of antitumor immune cells. A novel circular RNA, circSpdyA, encodes a 127‐amino acid peptide that directly binds fatty acid synthase (FASN) and promotes de novo fatty acid synthesis. This leads to transcriptional suppression of NK cell activation factors and impaired NK cell function [94]. However, the specific mechanism by which the 127‐AA peptide interferes with NK cell activation remains unclear. The accumulation of the bile acid metabolite isolithocholic acid (iso‐LCA) also leads to immunosuppression. Deletion of aldehyde‐ketone reductase family 1 member D1 (AKR1D1) increases the abundance of Bacteroides ovale, which converts deoxycholic acid to iso‐LCA. Accumulated iso‐LCA impairs the antitumor function of hepatic NK cells via a CREB1 phosphorylation‐dependent pathway. Notably, the potassium‐sparing diuretic spironolactone reverses iso‐LCA‐mediated immune escape and synergistically enhances anti‐PD‐1 efficacy in hepatocellular carcinoma [95].

Targeting dysregulated lipid metabolism pathways in the TME holds promise for reshaping the metabolic adaptability and activity of anti‐tumor immune cells, thereby enhancing the persistence and efficacy of immunotherapy. Inhibiting sphingolipid synthesis can restore IFN‐γ signaling, evade immune surveillance by NK and CD8^+^T cells, and amplify anti‐tumor immune responses [92]. Reports indicate that knocking out SREBP2 or combining membrane‐hardening agents with NK cell immunotherapy significantly enhances NK cell‐mediated killing activity against gastric cancer cells [93]. Additionally, to prevent CD8^+^T cells from differentiating into Tex cells, one potential strategy involves targeting type I interferon (IFN‐I). Chronic IFN‐I stimulation disrupts lipid metabolism and redox balance in Tex cells, leading to abnormal lipid accumulation and elevated oxidative stress [96]. Lipid peroxidation‐related oxidative stress also leads to NK cell dysfunction. However, activation of the Nrf2 antioxidant signaling pathway effectively reverses this process. Nrf2 activation not only restores NK cell metabolic capacity and cytotoxicity but also reprograms NK cells to acquire a metabolically flexible phenotype, enabling adaptation to nutrient‐depleted conditions within the tumor microenvironment. This metabolic adaptability allows NK cells to maintain potent antitumor activity in vivo [97]. Additionally, nanovaccines targeting lipid metabolism can be used to modulate the function of antitumor immune cells. For example, Qin et al. [98] developed a tumor‐infiltrating dendritic cell (TIDC)‐targeted in situ nanovaccine (TPOP) capable of modulating lipid metabolism. This vaccine enhances TIDCs' cross‐presentation capacity by inhibiting de novo fatty acid synthesis and reducing lipid accumulation. When combined with immune checkpoint inhibitor therapy, TPOP induces systemic antitumor responses and significantly suppresses distant tumor growth.

### 4.2 Regulatory Mechanisms and Therapeutic Strategies of Lipid Metabolism in Immunosuppressive Cells

Immunosuppressive cells are also affected by lipid metabolism, which significantly regulates the function of immunosuppressive cells [99]. Circulating monocyte‐derived TAMs in liver metastases highly express CD36, recruited via the CCL2/CCR2/p110γ signaling axis, and reprogram lipid metabolism. This leads to downregulation of sphingolipids, weakening lipid raft‐dependent p110γ activation, and consequently enhancing immunosuppressive functions. Targeting CD36 blocks monocyte recruitment and lipid metabolism reprogramming, reverses TAM‐mediated immunosuppression, inhibits liver metastasis, and provides a novel therapeutic target [100]. Additionally, fatty acid oxidation (FAO) metabolism also regulates TAM function. Studies indicate that IL‐34 enhances macrophage FAO via the CD36 receptor, driving the polarization of alternatively activated TAMs and suppressing CD8^+^T cell function, thereby mediating immune escape [101]. Similarly, overexpression of the immune checkpoint molecule VSIG4 in macrophages activates the JAK2/STAT3 pathway, enhances fatty acid oxidation (FAO), and upregulates PPAR‐γ expression, collectively promoting alternative‐activated TAM polarization and tumor progression [102]. Additionally, modulating endoplasmic reticulum stress in alternatively activated TAMs may offer a therapeutic target for immunotherapy. These TAMs exhibit strong chemotaxis toward hypoxic tumor regions, where activation of the IRE1‐XBP1 pathway inhibits glycolysis, enhances oxidative phosphorylation (OXPHOS), and promotes intracellular lipid accumulation, thereby sustaining their pro‐tumor phenotype [103]. Key enzymes in lipid metabolism are also closely associated with the differentiation and infiltration of immune‐suppressive cells. Sterol‐regulatory‐element‐binding proteins (SREBPs) exhibit significantly increased activity within tumor cells. SREBPs support Treg functional maturation by regulating SCAP to activate FASN‐mediated de novo fatty acid synthesis [104]. Lysosomal acid lipase (LAL), a key enzyme in neutral lipid metabolism, is essential for the normal maturation of myeloid cells. LAL deficiency drives myeloid cells to acquire an MDSC phenotype, characterized by enhanced glycolysis and glutamine catabolism, excessive reactive oxygen species production, and potent immune suppression, thereby accelerating tumor progression [105]. Acetyl‐CoA acetyltransferase 1 (ACAT1), a key enzyme in fatty acid oxidation and ketone body production, is significantly downregulated in infiltrating glioblastoma‐associated myeloid‐derived suppressor cells (MDSCs). Deficiency of ACAT1 in myeloid cells promotes macrophage secretion of CXCL1, stimulating MDSC expansion and aggregation. This enhances the immunosuppressive tumor microenvironment and promotes tumor growth [106]. In hepatocellular carcinoma, fatty acid‐binding protein 5 (FABP5) inhibits β‐oxidation in monocytes, leading to lipid droplet accumulation and activation of the PPARα‐IL‐10‐JNK‐STAT3 pathway. This pathway upregulates PD‐L1 on Treg cell surfaces and promotes immune tolerance [107]. Tumor‐derived granulocyte‐macrophage colony‐stimulating factor (GM‐CSF) induces MDSC expression of fatty acid transport protein 2 (FATP2) by activating the STAT3 pathway, thereby promoting lipid accumulation and reactive oxygen species production to enhance their immunosuppressive activity [108]. Therefore, targeting FATP2 offers a promising new avenue for enhancing the efficacy of anti‐PD‐L1 therapy. Furthermore, serine and palmitic acid enriched in the tumor microenvironment are converted into sphingosine via Sptlc2‐mediated sphingosine synthesis. This compound binds to the transcription factor c‐Fos, enhancing its recruitment to the PD‐1 promoter and thereby promoting PD‐1‐dependent Treg differentiation and immune suppression [109]. In cholangiocarcinoma, autocrine signaling of CXCL6 activates the CXCR1/2‐JAK‐STAT/PI3K pathway, inducing lipid metabolism reprogramming in TANs and activating the RAS/MAPK pathway, thereby promoting the formation of TAN extracellular networks (NETs). NETs subsequently block CD8^+^T cell infiltration, leading to resistance to immunotherapy [110].

Increasing evidence indicates that interventions targeting lipid metabolism can reverse immune suppression induced by immunosuppressive cells and inhibit tumor growth. In macrophages, VSIG4 correlates with TAM function and tumor progression; inhibiting VSIG4 or blocking fatty acid oxidation reverses the alternative activated polarization and disrupts the immunosuppressive microenvironment [102]. Research reports indicate that encapsulating the IRE1‐XBP1 inhibitor KIRA6 within α‐tocopherol‐based reductive nanoemulsions can restore glycolysis and inhibit FAO under hypoxic conditions. Furthermore, this nanomedicine simultaneously alleviates endoplasmic reticulum stress and oxidative stress, effectively reprogramming alternatively activated TAMs and enhancing tumor sensitivity to PD‐1 blockade. This provides a clinically significant strategy for overcoming immune therapy resistance [103]. Additionally, the lipid metabolism‐related enzyme acyl‐CoA δ‐isomerase 2 (ECI2) suppresses ether lipid synthesis by inhibiting alkane glycerol phosphate synthase (AGPS), thereby downregulating IL‐8 expression, reducing TAN recruitment and NET formation, and ultimately inhibiting colorectal cancer progression. highlighting the therapeutic potential of targeting the ether lipid‐neutrophil axis [111]. Research has revealed that melatonin induces pancreatic cancer cells to secrete CXCL2, recruiting neutrophils and reprogramming them into N1‐like antitumor phenotypes. Reprogrammed TANs exhibit enhanced fatty acid oxidation, increased reactive oxygen species production, and heightened NET formation. Through contact‐dependent cytotoxicity, they jointly trigger tumor cell apoptosis, thereby inhibiting tumor progression [112]. Additionally, dietary restriction of omega‐6 fatty acids or inhibition of arachidonic acid (AA) biosynthesis can reverse lipid metabolic reprogramming, restore treatment sensitivity, and alleviate TAN‐driven immunosuppression. In triple‐negative breast cancer, tumor cells accumulate lipid droplets and release AA‐rich extracellular vesicles that reprogram neutrophil lipid metabolism, thereby driving an immunosuppressive microenvironment and causing resistance to PD‐1‐combined chemotherapy [113].

In fact, lipid metabolism is not an isolated energy supply system but serves as a central regulatory hub for immune responses, playing a crucial role in immune cell phenotype conversion and functional maintenance. Therefore, targeting abnormal lipid metabolic pathways within the tumor microenvironment (TME) holds promise for reshaping the metabolic adaptability and antitumor activity of immune cells, thereby enhancing the durability and efficacy of immunotherapy. Most current research remains in the preclinical stage. Drugs targeting FASN (e.g. TVB‐2640) are undergoing clinical trials for antitumor therapy. However, large‐scale, multicenter clinical trial data remain relatively scarce, necessitating further validation of their safety and efficacy (Table 3). Additionally, fatty acid supplementation has been shown to inhibit tumor growth and overcome tumor resistance to immunotherapy [114, 115]. Therefore, dietary intervention with fatty acids offers a novel approach to tumor treatment by achieving systemic metabolic regulation. However, this therapeutic strategy also presents various challenges. For instance, due to the complex mechanisms of action of different fatty acids, uncertainty regarding intake levels, patient variability, and conflicting mechanisms in combination therapies, further research is required to develop appropriate, precise, and effective personalized dietary intervention protocols for fatty acids.

**TABLE 3:** Clinical trials of lipid metabolism drugs in cancer treatment.

Target		Drug	Mechanism	Targeted immune cells	Cancer types	Clinical trial	Combination therapy
Metabolism enzyme	FASN	TVB‐2640	Inhibiting FASN thereby suppresses fatty acid synthesis.	T cell	Prostatic Neoplasms	NCT05743621(I) Recruiting	Xtandi
					Prostate Cancer	NCT04337580 (II) Recruiting	Omeprazole
					Cancer of the Breast	NCT02595372(II) Completed	Omeprazole
					HER2+ Metastatic Breast Cancer	NCT03179904(II) Active, not Recruiting	Trastuzumab
					Metastatic Pancreatic Ductal Adenocarcinoma	NCT06608927(III) Active, not Recruiting	Chemotherapy

*Note*: Retrieved from https://clinicaltrials.gov/.

## 5 Regulatory Mechanisms and Therapeutic Strategies of Nucleotide Metabolism on Immune Cells

Many invasive behaviors of cancer cells, including proliferation, metastasis, and immune evasion, depend on enhanced nucleotide metabolism [116]. Nucleotide metabolism encompasses de novo synthesis, salvage synthesis, and extracellular nucleotide metabolism. Within extracellular nucleotide metabolism, the CD39/CD73 (extracellular nucleotidase)‐adenosine axis plays a pivotal role in immune suppression. Understanding nucleotide metabolic pathways in immune cells facilitates the development of cancer immunotherapy strategies targeting nucleotide metabolic reprogramming.

### 5.1 Regulatory Mechanisms and Therapeutic Strategies of Nucleotide Metabolism in Counteracting Anti‐tumor Immune Cells

Adenosine (ADO) suppresses CD8^+^T cell effector function by promoting Casitas B‐lineage lymphoma b (Cbl‐b)‐mediated degradation of Notch1. Conversely, restoration of Notch1 signaling significantly enhances CD8^+^T cell effector function, antitumor responses, and resistance to immunosuppression [117]. In TME, adenosine can also exert immunosuppressive effects in multiple immune cell types via cell surface receptors A2ARs/A2BR [118, 119]. Balanced nucleoside transporter‐1 (ENT1) is the primary regulator of extracellular adenosine concentration. ENT1‐mediated adenosine uptake inhibits phosphoadenylate synthase activity in activated T cells, thereby suppressing the production of uridine monophosphate and its derivatives. This, in turn, inhibits antitumor immunity and T‐cell pyrimidine synthesis. Blocking or knocking out host ENT1 significantly enhances CD8^+^T cell‐dependent antitumor responses [120]. Therefore, ENT1 represents a potential therapeutic target for overcoming extracellular adenosine‐induced immunosuppression and enhancing PD‐1 blockade activity. Overexpression of nucleoside diphosphate kinase 4 (NME4) correlates with reduced immune cell infiltration, particularly of CD8^+^T cells. NME4 promotes tumor immune escape by significantly suppressing CD8^+^T cell infiltration and activation [121]. Extracellular 5'‐nucleotidase (CD73) is a key enzyme regulator of adenosine production. Its abnormal expression on the cell surface leads to extracellular accumulation of adenosine, significantly suppressing anti‐tumor T cell function and enhancing the activity of immunosuppressive cells, thereby promoting tumor immune escape. Furthermore, overexpression of the CD73 gene has been observed in multiple cancers, correlating with poor response to immunotherapy and unfavorable prognosis [122].

The accumulation of immunosuppression in TME‐associated ADO promotes immune evasion and tumor progression. To alleviate this immunosuppression, therapeutic approaches targeting adenosine production or adenosine receptor signaling are under development. Cytokine interleukin‐7 (IL‐7) signaling can counteract the immunosuppressive effects of adenosine, thereby promoting the accumulation of tumor‐associated CD8^+^T cells in melanoma models [123]. DZD2269 is a novel A2AR antagonist capable of completely blocking A2AR‐mediated immunosuppression commonly observed in the tumor microenvironment (TME). Its primary mechanisms include inhibiting CREB phosphorylation in T cells, restoring Th1 cytokine secretion in peripheral blood mononuclear cells (PBMCs), and stimulating dendritic cell (DC) maturation [124]. Consequently, the clinical development of DZD2269 in cancer patients is warranted (NCT04634344). M1069 is a highly selective dual antagonist of A2AR and A2BR. Compared to selective A2AR antagonists, M1069 more effectively suppresses secretion of pro‐tumor cytokines CXCL1 and CXCL5, and rescues IL‐12 secretion function in adenosine‐differentiated dendritic cells. It counters the immunosuppressive mechanism of high‐concentration adenosine both in vitro and in vivo [125]. Additionally, Xu et al. [126] developed a novel core/shell nanostructure platform composed of mesoporous silica nanoparticles coated with macrophage membranes, capable of loading catalase, doxorubicin, and residual to enhance the efficacy of immunotherapy. The nanoplatform enhances CD8^+^T cell‐mediated immune responses by blocking A2AR and stimulating DCs, thereby improving the microenvironment. Chen et al. [127] discovered a novel ultrasound‐responsive liposome system (BFPL) that suppresses ADO production and enhances the efficacy of anti‐PD‐L1 immunotherapy. Furthermore, targeting CD73 represents a highly promising therapeutic strategy in cancer immunotherapy. Shin et al. [128] observed that the novel CD73 antibody (GI‐αCD73) effectively alleviated the functional defect in IL‐2 signaling within CD8^+^T cells under adenosine‐enriched conditions. However, under adenosine‐rich conditions, co‐administration of the novel bifunctional GI‐αCD73/IL‐2v fusion protein enhanced CD8^+^T cell activation and proliferation, inducing potent antitumor activity while maintaining favorable safety. Research has demonstrated that multifunctional gold@copper hypoxic selenium nanoparticles (ACS NPs) significantly suppress CD73 expression to attenuate ADO‐driven immunosuppression, thereby enhancing the infiltration and activity of antitumor T cells in glioblastoma [129]. This study reveals that regulating CD73‐mediated tumor immune suppression is a key strategy for improving the efficacy of immunotherapy in glioblastoma.

### 5.2 Regulatory Mechanisms and Therapeutic Strategies of Nucleotide Metabolism in Immunosuppressive Cells

Adenosine influences TAM polarization through A2AR and A2BR, thereby driving phenotypes that support tumor progression and immune evasion [130]. Additionally, in non‐small cell lung cancer, adenosine activates A2AR, leading to upregulation of CXCL5 in macrophages. CXCL5 also induces neutrophil extracellular trap release (NETosis). CD8^+^T cells treated with NETs exhibit exhaustion‐associated pathways. However, A2AR inhibition significantly downregulates CXCL5 expression and reduces neutrophil infiltration, thereby alleviating CD8^+^T cell dysfunction [131]. The enzyme cytidine deaminase in the pyrimidine recycling pathway is a key factor limiting the replenishment of uracil nucleotides in the extracellular environment. It recruits immunosuppressive TAMs by activating the uridine diphosphate receptor P2Y6 and impedes the recruitment and activation of CD8^+^T cells, ultimately leading to the failure of anti‐PD‐1 therapy [132]. CD73 autonomously expressed by tumor cells promotes Treg recruitment, with CCL5 identified as a key downstream effector molecule of CD73. CD73 activates the p38‐STAT1 axis through the tumor cell‐autonomous adenosine‐A2AR signaling pathway, transcriptionally upregulating CCL5 to recruit Tregs to pancreatic tumors and establish an immunosuppressive microenvironment [133]. Tumor‐associated glycosphingolipid—spherical H ceramide (GHCer)—promotes Treg differentiation by activating the A2AR/cAMP/PKA pathway. Furthermore, GHCer enhances Treg inhibitory capacity by upregulating suppressor molecules such as Lymphocyte Activation Gene 3, Cytotoxic T Lymphocyte‐Associated Protein 4, and Programmed Cell Death Ligation 1. Additionally, GHCer‐induced Tregs express CD39 and CD73, further enhancing adenosine production [134]. Multiple mechanisms collectively establish an immunosuppressive tumor microenvironment.

Targeting the adenosine signaling pathway enhances the efficacy of immunotherapy, offering a promising strategy to overcome drug resistance. The A2AR antagonist (SCH58261), an immunometabolic checkpoint inhibitor, has been found to alleviate adenosine‐related immunosuppression, reduce Treg recruitment, enhance antitumor immune responses, and induce immune memory responses [135]. AMD3100 (CXCR4 antagonist) and CPI‐444 (A2AR inhibitor) synergistically enhance antitumor responses by reducing tumor‐associated macrophages, myeloid‐derived suppressor cells, and modulating T cells. This combination promotes ICD induction, dendritic cell maturation, and alleviation of the immunosuppressive microenvironment, significantly prolonging survival in glioblastoma mouse models [136]. Research has found that compared to anti‐PD‐L1 monotherapy, CD73 inhibition can slow tumor progression and exhibit synergistic effects with anti‐PD‐L1. This combination regimen produces positive systemic immunomodulatory effects, including a decrease in the neutrophil‐to‐lymphocyte ratio and an increase in circulating CD4 and CD8^+^T cells [137]. Additionally, blocking STAT3 reduces CD39/CD73 expression and adenosine synthesis, and inhibits the differentiation of monocytes into MDSCs in vitro [138]. Scolaro et al. [132] demonstrated in drug‐resistant tumor models that inhibiting CDA in cancer cells (or P2Y6 in TAMs) via pharmacological or genetic approaches disrupts TAM‐mediated immune suppression, promotes cytotoxic T cell infiltration, and enhances sensitivity to anti‐PD‐1 therapy.

Nucleotide metabolism dynamically participates in the cancer‐immunity cycle, primarily exerting antitumor effects by mediating immune cell functions. Notably, compared to other immune cells, systematic studies on nucleotide metabolic programs in DCs, NK cells, and CAFs remain limited. The mechanisms by which nucleotide metabolism regulates DCs, NK cells, and CAFs, as well as the induction of key subset‐specific differences and functional outcomes, remain to be further elucidated. Targeting nucleotide metabolic pathways in combination with immunotherapy drugs holds promise for achieving synergistic effects across multiple cancers. Currently, CD73 inhibitors, CD39 antagonists, and adenosine receptor antagonists are all in critical clinical development stages for tumor therapy. Some of these drugs have demonstrated efficacy in combination therapies, with particularly positive results in non‐small cell lung cancer, pancreatic cancer, and renal cell carcinoma (Table 4). Future efforts should focus on further optimizing drug selectivity, exploring combination therapy strategies, and establishing biomarker‐guided patient stratification to achieve precision medicine.

**TABLE 4:** Clinical trials of nucleotide metabolism drugs in cancer treatment.

Target		Drug	Mechanism	Targeted immune cells	Cancer types	Clinical trial	Combination therapy
Metabolism enzyme	CD39	ES002023	Targeting CD39 to inhibit adenosine production and maintain pro‐inflammatory ATP levels	T cell, DC, NK, Treg	Advanced Solid Tumor	NCT05075564 (I) Completed	
		TTX‐030			Solid Tumor, Adult	NCT04306900 (I/Ib) Completed	Pembrolizumab or Budigalimab and/or Chemotherapy
					Pancreatic Cancer	NCT06119217 (II) Active, not recruiting	Budigalimab
					Solid Tumor, Lymphoma	NCT03884556 (I) Completed	Pembrolizumab or Chemotherapy
		IPH5201			Advanced Solid Tumor	NCT04261075 (I) Completed	Durvalumab±Oleclumab
					Non‐Small Cell Lung Cancer	NCT05742607 (II) Recruiting	Durvalumab
		SRF617			Advanced Solid Tumor	NCT04336098 (I) Completed	None
		ES014	Simultaneously targeting CD39 and TGF‐β to remodel the immunosuppressive state within the tumour microenvironment		Advanced Solid Tumor	NCT06543056 (II) Recruiting	None
					Advanced Solid Tumor	NCT05717348 (I) Active, not recruiting	None
	CD73	Oleclumab	Inhibit the enzymatic activity of CD73, block the production of adenosine, thereby relieving the immune suppression mediated by adenosine	CD8+T, Treg, MDSC	Carcinoma, Non‐Small‐Cell Lung	NCT03381274 (I/II) Active, not recruiting	None
		CPI‐006			Non‐Small Cell Lung Cancer, Renal Cell Cancer, Colorectal Cancer, Triple Negative Breast Cancer, Cervical Cancer, Ovarian Cancer, Pancreatic Cancer, Endometrial Cancer, Sarcoma, Squamous Cell Carcinoma of the Head and Neck, Bladder Cancer, Metastatic Castration Resistant Prostate Cancer, Non‐hodgkin Lymphoma	NCT03454451 (I) Completed	Ciforadenant or Pembrolizumab
		TJ004309			Advanced Solid Tumor	NCT04322006 (I/II) Active, not Recruiting	Toripalimab
					Ovarian Cancer, Head and Neck Cancer, Non‐Small Cell Lung Cancer, Gastrointestinal Cancer, Triple Negative Breast Cancer, Ovarian Carcinoma	NCT05001347(II) Completed	Atezolizumab
		AK119			Advanced or Metastatic Solid Tumors	NCT04572152 (1a/1b) Completed	AK104
					Solid Tumor	NCT05173792 (I) Completed	None
		IBI325			Solid Tumor	NCT05119998 (I) Completed	Sintilimab
		AB680 (Quemliclustat)			Advanced Pancreatic Cancer	NCT04104672 (I) Active, not Recruiting	zimberelimab (AB122), nab‐paclitaxel, gemcitabine
					Biliary Tract Carcinoma, Cholangiocarcinoma	NCT06048133 (II) Recruiting	Gemcitabine, Cisplatin, Zimberelimab
					Metastatic Pancreatic Ductal Adenocarcinoma	NCT06608927(III) Active, not Recruiting	Chemotherapy
		Mavrostobart(PT199)			Non‐Small Cell Lung Cancer, Pancreatic Ductal Adenocarcinoma	NCT05431270 (I/II) Recruiting	Checkpoint Inhibitor TreatinG
		IPH5301			Metastatic Breast Cancer, Metastatic Pancreatic Cancer, Metastatic Gastric Cancer, Metastatic Lung Cancer, Metastatic Ovary Cancer, Oesophageal Cancer, Endometrial Cancer	NCT05143970 (I) Recruiting	Chemotherapy, Trastuzumab
		ORIC‐533			Relapsed or Refractory Multiple Myeloma	NCT05227144 (I) Completed	None
		LY3475070			Advanced Cancer	NCT04148937(I) Completed	Pembrolizumab
		Sym024			Metastatic Cancer, Solid Tumor	NCT04672434 (I) Completed	Sym021
		AK137	Bispecific antibody targeting CD73 and LAG‐3		Advanced Malignant Tumors	NCT06691360 (I) Not yet recruiting	None
		JAB‐BX102	Targeting CD73 and blocking adenosine production		Solid Tumor	NCT05174585 (1/2a) Completed	Pembrolizumab
		GI‐108	Targeting CD73 and IL2RA to simultaneously regulate the immunosuppressive mechanisms in the tumor microenvironment		Non‐small Cell Lung Cancer,Head and Neck, Pancreatic Cancer, Renal Cell Carcinoma	NCT07172802(I/II) Recruiting	None
Signal molecules	Adenosine	AZD4635	Targeting A2AR to reverse the immunosuppressive state in the tumor microenvironment	T cell, NK, CTL	Prostate Cancer, Metastatic Castration‐Resistant Prostate Cancer	NCT04089553 (II) Completed	None
					Advanced Solid Malignancies, Non‐Small Cell Lung Cancer, Metastatic Castrate‐Resistant Prostate Carcinoma, Colorectal Carcinoma	NCT02740985 (I) Completed	None
					Progressive Metastatic Castrate‐Resistant Prostate Cancer	NCT04495179 (I/II) Completed	Durvalumab, Cabazitaxel

*Note*: Retrieved from https://clinicaltrials.gov/.

## 6 Regulatory Mechanisms and Therapeutic Strategies of Other Metabolism on Immune Cells

### 6.1 Regulatory Mechanisms and Therapeutic Strategies of Other Metabolism in Counteracting Anti‐tumor Immune Cells

Mitochondrial loss or dysfunction is a central component of tumor metabolic reprogramming. Tumor cells can drive metabolic restructuring through mitochondrial dysfunction, which not only satisfies their own rapid growth demands but also suppresses anti‐tumor immune cells, thereby achieving immune escape [139]. Mitochondrial damage leads to elevated reactive oxygen species (ROS) levels, which in turn induce CD8^+^T cell exhaustion [140]. Research indicates that inhibiting RE1α reduces mitochondrial ROS levels in tumor‐infiltrating CD8^+^T cells, reverses T cell senescence, and enhances the efficacy of immunotherapy [141]. Repolarizing alternatively activated TAMs to a pro‐inflammatory activated phenotype has gained increasing recognition as an effective strategy against refractory malignancies. ROS activates the ATM‐Chk2 DNA damage response pathway, inducing Chk2‐dependent PKM2 phosphorylation (at sites T95/T195), enhancing glycolysis, and promoting p21‐mediated cell cycle arrest, thereby driving macrophage polarization toward pro‐inflammatory activated TAMs [142]. Bezafibrate (BEZ) acts as a potent regulator of mitochondrial biogenesis by activating the PGC‐1α/PPAR complex to modulate lipid metabolism and fatty acid oxidation. Pan et al. [143] synthesized a mitochondrial‐targeted BEZ analog (Mito‐BEZ), which significantly enhances CD8^+^T cell mitochondrial function while inhibiting oxidative metabolism in cancer cells. Mito‐BEZ thereby activates T cell immune responses within the tumor microenvironment and exerts a marked inhibitory effect on lung cancer progression and metastasis. Exogenous mitochondrial delivery to CD8^+^T cells represents an emerging therapeutic strategy [144]. An innovative technology enables intercellular transfer of mitochondria from bone marrow mesenchymal cells to CD8^+^T cells by establishing nanotube connections between the two cell types. Recipient T cells consequently exhibit enhanced metabolic adaptability and antitumor activity [145].

The tricarboxylic acid cycle (TCA cycle), also known as the citric acid cycle, serves as the central hub of cellular respiration and energy metabolism. The accumulation of immune signaling metabolites within the TCA cycle can similarly suppress the function of anti‐tumor immune cells. Succinate, an intermediate product of the TCA cycle, accumulates in tumors harboring mutations in succinate dehydrogenase (SDH). Studies have revealed that in tumors lacking the succinate dehydrogenase B subunit (SDHB), succinate accumulation enhances mitochondrial function via Bcl‐2/E1B 19 kDa protein 3 (BNIP3)‐mediated mitochondrial autophagy, thereby promoting CD8^+^T cell antitumor activity. It also modulates epigenetic regulation to enhance expression of genes associated with stem cell characteristics, promotes memory T cell generation, and exhibits synergistic effects when combined with immune checkpoint inhibitors [146]. Conversely, succinate secreted by tumor cells can activate the succinate receptor (SUCNR1) pathway, thereby stabilizing PI3K/HIF1α signaling transmission. This process polarizes resident macrophages into TAMs, promoting cancer cell migration [147]. Recent studies indicate that tumor‐derived microsomes (SMPs) carrying succinate can reprogram macrophage metabolism. SMPs enhance glycolysis and suppress the tricarboxylic acid cycle through succinylation‐dependent mechanisms, thereby promoting pro‐inflammatory activation and TAM polarization [148]. This discovery reveals translational opportunities for immunotherapy based on post‐transplant metabolic regulation. Another class of metabolites, such as fumaric acid (also known as fumaric acid) and 2‐hydroxyglutaric acid (2‐HG). Deficiency or inactivation of fumarate hydratase (FH) leads to fumarate accumulation in tumor interstitial fluid. Elevated fumarate directly succinylates ZAP70 at cysteine residues C96 and C102, thereby inhibiting CD8^+^T cell activation and antitumor immunity [149]. Additionally, acquired functional mutations in isocitrate dehydrogenase (IDH) generate D‐2‐hydroxyglucose, which, when taken up by CD8^+^T cells, alters glycolysis and impairs their cytotoxic function [150]. In contrast, Tex cells depleted within tumors exhibit impaired mitochondrial function and reduced L‐2‐HG levels. Treatment of Tex cells with L‐2‐HG improves mitochondrial metabolism, decreases H3K27me3 abundance, and promotes memory T cell differentiation [151]. Therefore, L‐2‐HG supplementation holds promise as an effective strategy to restore CD8^+^T cell antitumor activity. Furthermore, macrophage‐derived itaconic acid promotes hepatocellular carcinoma progression by inducing Eomesodermin‐mediated CD8^+^T cell exhaustion through epigenetic mechanisms [152]. Excess itaconic acid also inhibits antigen presentation by DCs, impairs T‐cell responses, and promotes immune escape and tumor progression. During anti‐PD‐1 therapy, IFN‐γ released by CD8^+^T cells upregulate ACOD1 expression in macrophages via the JAK‐STAT1 pathway, thereby increasing itaconic acid production. Therefore, targeting ACOD1 or neutralizing succinic acid lactone can disrupt this negative feedback loop, restore DC function, and synergistically enhance antitumor immunity with anti‐PD‐1 therapy, providing a potential target for overcoming immune therapy resistance [153, 154].

### 6.2 Regulatory Mechanisms and Therapeutic Strategies of Other Metabolism in Immunosuppressive Cells

Efficient mitochondrial OXPHOS is essential for maintaining the energy homeostasis and suppressive capacity of immune‐inhibitory cells. YAP enhances mitochondrial protein translation and OXPHOS in tumor‐infiltrating regulatory T cells (TI‐Tregs) by upregulating Lars2, thereby sustaining their immunosuppressive function. Combined YAP inhibition with a leucine‐restricted diet disrupts Lars2‐dependent mitochondrial function, weakens TI‐Treg activity, and inhibits tumor progression [155]. Conversely, NAC1 deficiency stabilizes Foxp3 expression, enhances CD36‐mediated lipid uptake, and promotes PGC‐1α‐regulated mitochondrial metabolism, thereby enhancing Treg adaptability and suppressive efficacy within acidic, nutrient‐depleted tumor microenvironments [156]. Notably, balancing glycolysis with OXPHOS rather than solely inhibiting glycolysis provides a more potent immunomodulatory strategy: enhancing OXPHOS not only reduces Treg and MDSC activity but also promotes CD8^+^/CD4^+^ T cell infiltration and IFN‐γ secretion. When combined with PD‐L1 silencing, it yields superior antitumor effects [157]

Hypoxia‐immune cell interactions are one of the key factors determining the complexity of immune suppression in the tumor microenvironment. The hypoxic environment promotes Treg infiltration, suppresses NK cell cytotoxicity, recruits MDSCs, and maintains their inhibitory functions, thereby reducing the responsiveness to immunotherapy [158, 159, 160]. HIF1α plays a crucial role in this process. MLL3 deletion stabilizes HIF1α, increases tumor secretion of CCL2, and promotes Treg recruitment and immune suppression [161]. Additionally, HIF1α expression correlates with MDSC function [162]. Hypoxia‐induced HIF1α upregulates the macrophage adhesion molecule ALCAM, which spatially colocalizes with CD8^+^T cells, accelerating their exhaustion and leading to immunotherapy resistance. Inhibiting HIF1α downregulates ALCAM expression, reverses T cell exhaustion, and enhances antitumor immunity, offering a novel strategy to overcome hypoxia‐associated immunotherapy resistance [163]. Compared to normoxic CAFs, hypoxic CAFs exhibit stronger capacity to suppress CTL cytotoxicity [164]. Hypoxia upregulates CAF secretion of multiple immunosuppressive factors, including TGF‐β, IL‐6, IL‐10, VEGF, and PD‐L1, thereby further enhancing immune evasion effects [164]. Hypoxia induces CAF expression and secretion of POSTN, which promotes colorectal cancer cell migration and proliferation [165]. CAF‐derived CCL5 also enhances hepatocellular carcinoma metastasis via the HIF1α‐ZEB1 axis [166]. Therefore, targeting HIF1α to alleviate hypoxia may prevent immune escape and enhance therapeutic response. For instance, the traditional Chinese medicine extract YYFZBJS downregulates the HIF1α signaling pathway, inhibits peripheral Treg differentiation, and blocks STAT3/NF‐κB/MMP‐related tumorigenesis pathways, thereby reshaping the immune microenvironment and suppressing colorectal cancer development [167]. Combination therapy with HIFα and Treg inhibitors enhances antitumor immunity in mice [168]. Li et al. [169] developed an immunomodulatory nanoplatform (FEM@PFC) that alleviates tumor hypoxia by delivering oxygen via perfluorocarbon while simultaneously inhibiting Treg infiltration, depleting glutathione, and downregulating Foxp3 expression and its suppressive function. This formulation synergistically enhances radiotherapy‐induced immunogenic cell death and dendritic cell maturation, thereby reactivating effector T cell responses.

## 7 Summary and Prospect

Tumor cells satisfy their rapid proliferation, and survival demands through multiple metabolic pathways, such as glycolysis, amino acid metabolism, and lipid metabolism. These pathways do not exist in isolation but are interconnected and mutually complementary. Through intricate interactions, these metabolic pathways collectively shape a TME that profoundly influences immune responses. Key metabolites and signaling pathways cross‐regulate each other, jointly contributing to immune evasion [170]. For instance, lactic acid accumulation from glycolysis in tumor cells suppresses CD8^+^T cell and NK cell activity while promoting Treg differentiation and function, thereby creating an immunosuppressive microenvironment [171]. Similarly, under hypoxic/acidic conditions, tumor cells upregulate LDH5 via HIF1α, driving a hyperglycolytic state and massive ATP release. Concurrently, hypoxia and acidity induce overexpression of CD39/CD73 in cancer and stromal cells, rapidly metabolizing ATP into adenosine. This directly suppresses effector T cell function and promotes infiltration of inhibitory immune cells like FOXP3+ Tregs [172]. Similarly, key enzymes in the tryptophan metabolic pathway‐IDO1 or TDO‐deplete tryptophan in the microenvironment while accumulating products like kynurenine. This simultaneously induces T cell apoptosis, promotes Treg differentiation, and suppresses dendritic cell function [173, 174]. By synthesizing these findings, several shared principles emerge that govern metabolic immune evasion. First, nutrient competition is a common theme, where tumor cells outcompete immune cells for essential fuels like glucose and glutamine, leading to a state of starvation that impairs effector functions. Second, the accumulation of immunoregulatory metabolites such as lactate, adenosine, and kynurenine creates a profoundly immunosuppressive milieu, impairing effector lymphocytes while promoting the activity of regulatory subsets. Third, immune subsets exhibit context‐dependent metabolic adaptation, where anti‐tumor cells are often functionally crippled by nutrient restriction, whereas immunosuppressive cells like Tregs and TAMs are equipped to thrive in and even exploit the same hostile metabolic environment. Finally, pathway‐level crosstalk creates a complex and reinforcing network; for instance, hypoxia (HIF1α) not only drives glycolysis and lactate production but also induces the expression of CD39/CD73, which together amplify adenosine‐mediated suppression. Interactions among tumor metabolic pathways profoundly influence immune cell function through mechanisms such as nutrient competition, metabolite regulation, and immune cell adaptation, thereby promoting tumor immune escape. Deepening our understanding of these interactive mechanisms will aid in developing novel therapeutic strategies to overcome immune therapy resistance and enhance tumor treatment efficacy.

Tumor cell metabolism plays a pivotal role in reshaping the tumor immune microenvironment, suppressing anti‐tumor immune activation, and creating an immunosuppressive environment. Modulating tumor cell metabolic homeostasis to enhance anti‐tumor immune responses represents a highly promising therapeutic direction for the future. However, the complex interactions between tumor cell metabolism and immune regulation require further elucidation. Compounded by the significant heterogeneity of tumors and the diversity of pathogenic mechanisms among individuals, developing personalized therapeutic strategies that integrate metabolic and immune interventions remains a long‐term challenge. As tumor treatment enters the era of precision medicine, the clinical application of combination strategies targeting both metabolism and immunity must address several key challenges. First, it is essential to elucidate tumor‐type‐specific metabolic vulnerabilities. Future research should prioritize identifying which metabolic pathways are dominant in different cancers, enabling the selection of precise targeting strategies. Second, the context‐dependent effects of metabolic drugs on different immune cells must be characterized, as an intervention might boost anti‐tumor T cells while inadvertently supporting Tregs. Third, strategies to overcome compensatory metabolic pathways are critical, as tumor cells and immune cells can often bypass a single blocked route. Fourth, biomarker‐guided patient stratification, based on gene mutations, expression profiles, or even pre‐treatment metabolite levels, is essential to identify patients who are most likely to benefit from specific metabolic‐immune combination therapies. Finally, the safety and selectivity of metabolism‐targeted drugs must be rigorously evaluated, as many of these pathways are fundamental to normal cell function. These insights provide a rational basis for developing precision strategies in combined metabolic and immunotherapeutic interventions, ultimately aiming to improve the objective response rate (ORR) of treatment. This is a key factor in advancing the clinical application of this strategy. In addition to the above challenges, we are also faced with the fact that most metabolic drugs (e.g., inhibitors of ARG, FASN, IDO, and CD73) are still in preclinical or early clinical stages. Although preliminary evidence has confirmed the safety and efficacy of their combination with immune checkpoint inhibitors, most metabolic drugs remain at preclinical or early clinical stages. However, limitations such as compensatory pathway bypassing, insufficient drug accumulation, off‐target effects, drug resistance, and toxicity associated with single‐target metabolic pathways have created a critical bottleneck in the development of metabolic‐immune combination therapies, resulting in a scarcity of effective drugs [175, 176, 177]. However, with the advancement of modern technological revolutions, the combined application of multi‐omics, single‐cell, spatial omics, and real‐time metabolic imaging technologies enables in‐depth analysis of metabolic mechanisms, optimization of drug delivery systems, and development of precision treatment strategies. This approach has opened new avenues for tumor immunotherapy and pioneered novel frontiers in cancer treatment.

## Author Contributions

X.L., Z.D., and Y.Z. designed the manuscript. G.X., Z.Z., and X.J. wrote the manuscript with inputs from X.L., Z.D., and Y.Z. All authors read and approved the final manuscript.

## Funding

This study was supported by the Shanghai Natural Science Foundation (Grant No. 25ZR1402579) and the National Natural Science Foundation of China (Grant No. 82503230), Liaoning Provincial Science and Technology Program Joint Plan (Key R&D Program Projects) (Grant No. 2025JH2/101800164), and Liaoning Provincial Natural Science Foundation (Grant No. 2023‐MSLH‐141).

## Conflicts of Interest

The authors declare no conflicts of interest.

## Data Availability

The data that support the findings of this study are available on request from the corresponding author. The data are not publicly available due to privacy or ethical restrictions.
